# The Deubiquitinating Enzyme Cylindromatosis Dampens CD8^+^ T Cell Responses and Is a Critical Factor for Experimental Cerebral Malaria and Blood–Brain Barrier Damage

**DOI:** 10.3389/fimmu.2017.00027

**Published:** 2017-02-01

**Authors:** Ursula Schmid, Werner Stenzel, Josephin Koschel, Maria Raptaki, Xu Wang, Michael Naumann, Kai Matuschewski, Dirk Schlüter, Gopala Nishanth

**Affiliations:** ^1^Institute of Medical Microbiology and Hospital Hygiene, Otto-von-Guericke University Magdeburg, Magdeburg, Germany; ^2^Department of Neuropathology, Charite, Berlin, Germany; ^3^Institute of Experimental Internal Medicine, Otto-von-Guericke University Magdeburg, Magdeburg, Germany; ^4^Parasitology Unit, Max Planck Institute for Infection Biology, Berlin, Germany; ^5^Department of Molecular Parasitology, Humboldt University, Berlin, Germany; ^6^Organ-Specific Immune Regulation, Helmholtz Centre for Infection Research, Braunschweig, Germany

**Keywords:** malaria, deubiquitinating enzymes, CYLD, T cells, blood–brain barrier

## Abstract

Cerebral malaria is a severe complication of human malaria and may lead to death of *Plasmodium falciparum*-infected individuals. Cerebral malaria is associated with sequestration of parasitized red blood cells within the cerebral microvasculature resulting in damage of the blood–brain barrier and brain pathology. Although CD8^+^ T cells have been implicated in the development of murine experimental cerebral malaria (ECM), several other studies have shown that CD8^+^ T cells confer protection against blood-stage infections. Since the role of host deubiquitinating enzymes (DUBs) in malaria is yet unknown, we investigated how the DUB cylindromatosis (CYLD), an important inhibitor of several cellular signaling pathways, influences the outcome of ECM. Upon infection with *Plasmodium berghei* ANKA (*Pb*A) sporozoites or *Pb*A-infected red blood cells, at least 90% of *Cyld*^−/−^ mice survived the infection, whereas all congenic C57BL/6 mice displayed signatures of ECM, impaired parasite control, and disruption of the blood–brain barrier integrity. *Cyld* deficiency prevented brain pathology, including hemorrhagic lesions, enhanced activation of astrocytes and microglia, infiltration of CD8^+^ T cells, and apoptosis of endothelial cells. Furthermore, *Pb*A-specific CD8^+^ T cell responses were augmented in the blood of *Cyld*^−/−^ mice with increased production of interferon-γ and granzyme B and elevated activation of protein kinase C-θ and nuclear factor “kappa light-chain enhancer” of activated B cells. Importantly, accumulation of CD8^+^ T cells in the brain of *Cyld*^−/−^ mice was significantly reduced compared to C57BL/6 mice. Bone marrow chimera experiments showed that the absence of ECM signatures in infected *Cyld*^−/−^ mice could be attributed to hematopoietic and radioresistant parenchymal cells, most likely endothelial cells that did not undergo apoptosis. Together, we were able to show that host deubiqutinating enzymes play an important role in ECM and that CYLD promotes ECM supporting it as a potential therapeutic target for adjunct therapy to prevent cerebral complications of severe malaria.

## Introduction

Proteolytic removal of ubiquitin and ubiquitin-like modifications on key target proteins in signaling pathways permits rapid responses to environmental cues (1). A central regulatory pathway in innate and adaptive immunity is controlled by the nuclear factor “kappa light-chain enhancer” of activated B cells (NF-κB), which is essential for inflammatory responses and pathogen defense (2). For termination of inflammation, NF-κB needs to be inactivated through the activity of deubiquitinating enzymes (DUBs) such as A20 and CYLD (3). Cylindromatosis (turban tumor syndrome), also called CYLD, was originally identified as tumor suppressor gene, a mutation of which causes tumors of the hair follicles (cylindromas) (4, 5). CYLD is a DUB and negatively regulates activation of NF-κB, mitogen-activated protein kinases, and type I interferon (IFN) production by specifically cleaving lysine 63-linked polyubiquitin chains from several signaling molecules (6), including transforming growth factor beta-activated kinase 1, tumor necrosis factor (TNF) receptor-associated factors (TRAF) 2, TRAF6, B cell lymphoma 3, NF-κB essential modulator, retinoic acid-inducible gene 1, receptor-interacting serine/threonine kinase 2 (RIPK2), and signal transducer and activator of transcription 3 (STAT3) (7–12), thereby regulating non-degradative functions like protein trafficking, protein–protein interaction, and signal transduction.

Very few studies have addressed the role of CYLD in infectious diseases. *Cyld*^−/−^ mice infected with *Haemophilus influenzae* and *Escherichia coli* develop hyperinflammation due to a strong activation of the NF-κB signaling pathway (13, 14). On the contrary, *CYLD* deficiency protects mice from pneumolysin-induced acute lung injury and lethality (15). Previously, we have shown that CYLD inhibits IL-6 production by macrophages, which in turn induces STAT3-dependent protective fibrin production by hepatocytes in listeriosis (11). In addition, we could also show that CYLD impaired antilisterial immune responses in macrophages by deubiquitinating the kinase RIPK2 (12). However, how CYLD influences the course of parasitic infections remains entirely unknown.

Cerebral malaria is one of the most severe complications caused by infection with *Plasmodium falciparum* with fatality rates up to 25% (16). Brain pathology includes cerebral bleeding, brain edema, seizures, coma and, ultimately, death (17, 18). Experimental cerebral malaria (ECM), the rodent disease model of human cerebral malaria, is a widely used surrogate model to study the pathogenesis of cerebral malaria (19–21). A hallmark of cerebral malaria is the sequestration of *Plasmodium*-infected red blood cells on the brain vascular endothelium (22–24). Other characteristic features include activation of endothelial cells (25), production of proinflammatory cytokines and chemokines (26, 27), and disruption of the blood–brain barrier integrity (28). CD8^+^ T cells are known to play an important role in the pathology of ECM (29–31). CD8^+^ T cells mediate the disruption of the blood–brain barrier by fostering leukocyte accumulation (32) and by inducing apoptosis of endothelial cells through granzyme B and perforin-mediated cytotoxicity (29, 30). However, recent reports have suggested a protective role of CD8^+^ T cells against blood-stage malaria. CD8^+^ T cells eliminate *Plasmodium*-infected red blood cells and also modulate the immune response through production of cytokines (33–35).

Currently, the functional relevance of DUBs in human and ECM is completely unknown. In this study, we describe that *Cyld* expression in the hematopoietic and parenchymal cells lethally aggravated ECM, whereas *Cyld*-deficient mice were protected. Furthermore, we uncovered a dual function of CYLD in the pathogenesis of ECM: (i) CYLD impaired activation of PKC-θ and NF-κB in CD8^+^ T cells and (ii) CYLD promoted apoptosis of endothelial cells, thereby augmenting brain pathology and preventing survival during ECM.

## Results

### CYLD Is Essential for the Development of *Plasmodium berghei* ANKA (*Pb*A)-Induced ECM

**Figure 1:**
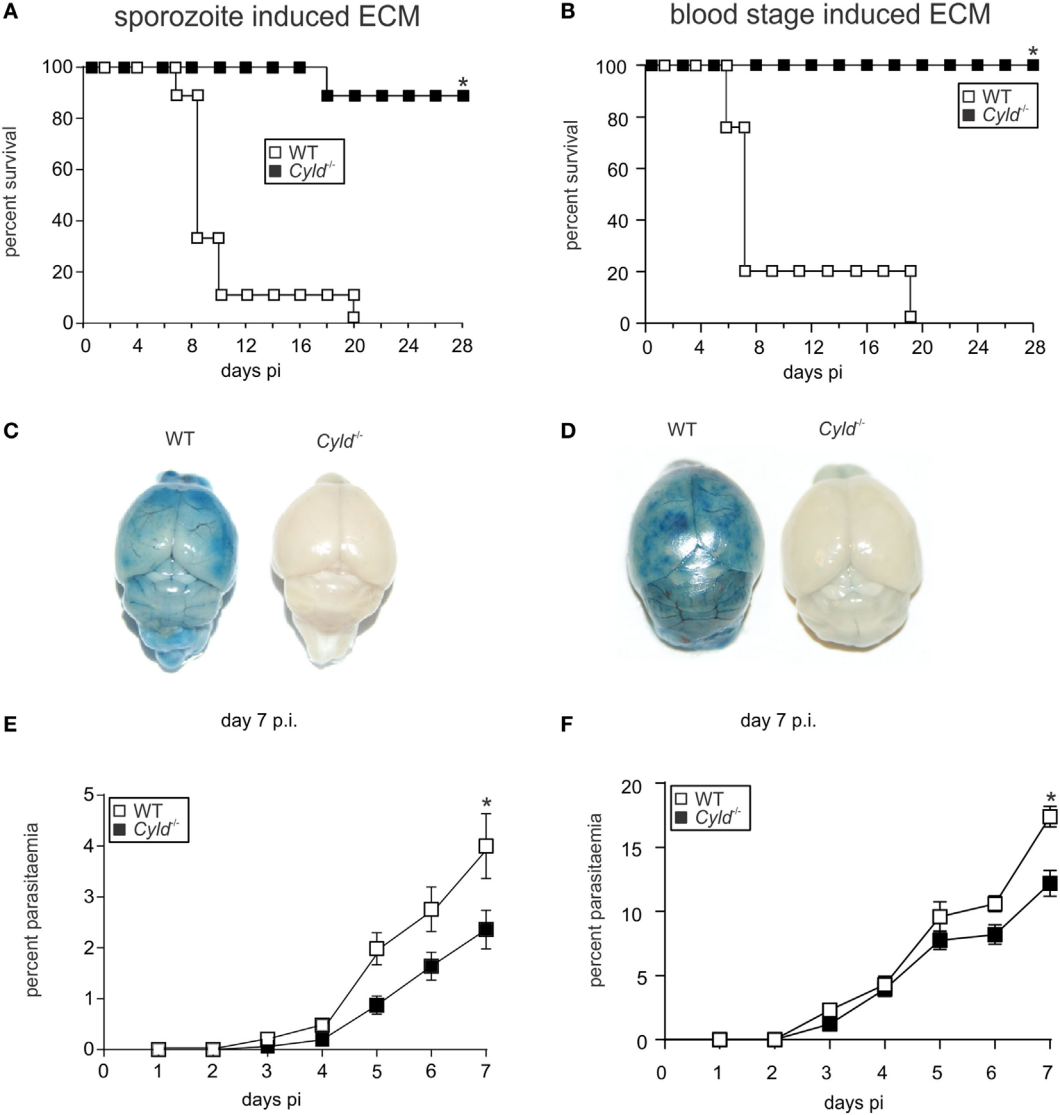
**Cyld^−/−^ mice are protected from Plasmodium berghei ANKA (*Pb*A)-induced experimental cerebral malaria**. **(A)** C57BL/6 *Cyld*^−/−^ and WT (*n* = 10 each) mice were intravenously (i.v.) infected with 20,000 *Pb*A sporozoites, and survival rates were monitored daily up to day 28 postinfection (p.i.). Data represent one of two independent experiments. **(B)** C57BL/6 *Cyld*^−/−^ and WT (*n* = 10 each) mice were intraperitoneally infected with 1 × 10^6^ *Pb*A-infected red blood cells, and the survival was monitored daily up to day 28 p.i. Data represent one of two independent experiments, *p* < 0.05 (log-rank test). **(C,D)** At day 7 p.i., Evans Blue was injected i.v. in mice infected with *Pb*A sporozoites **(C)** and *Pb*A-parasitized red blood cells **(D)**. The mice were euthanized 1 h later and perfused with saline, and the isolated brains were photographed. Representative images from three independent experiments are shown. **(E,F)** The percentage of parasitized erythrocytes in the peripheral blood was enumerated daily from Giemsa-stained thin blood smears after infection with *Pb*A sporozoites **(E)** and *Pb*A-infected red blood cells **(F)**, *p* < 0.05 (two-tailed Student’s *t*-test).

To study the function of CYLD in ECM, WT and *Cyld*^−/−^ mice were infected intravenously (i.v.) with 20,000 *Pb*A sporozoites. WT mice developed signature ECM symptoms, such as ataxia, loss of grip strength, and progressive paralysis, within 7 days postinfection (p.i.) (Figure 1A). In contrast, 90% of the infected *Cyld*^−/−^ mice survived without showing any symptoms of ECM. Similarly, on intraperitoneal (i.p.) infection with 10^6^ *Pb*A-infected red blood cells, all *Cyld*^−/−^ mice were protected from *Pb*A blood stage-induced ECM (Figure 1B). The brains of *Pb*A-infected WT mice displayed extensive blood–brain barrier damage as shown by Evans blue distribution, while the brains of infected *Cyld*^−/−^ mice showed no vascular leakage (Figures 1C,D), illustrating that CYLD is essential for the development ECM. A secondary lethal outcome of *Pb*A infection in WT mice is anemia around day 20 after infection (Figures 1A,B). In contrast to infected WT mice that did not survive beyond day 20, severe complications from parasite-induced anemia were not observed in infected *Cyld*^−/−^ mice.

On infection with blood-stage *Pb*A, parasitemia was detectable in WT and *Cyld*^−/−^ mice at day 3 p.i., and parasitemia progressively increased in both mouse strains (Figure 1E). However, the parasite burden was significantly reduced in *Cyld*^−/−^ mice at day 7 p.i. Similarly, on sporozoite infection, parasitemia increased in both strains of mice from day 3 p.i. onward (Figure 1F). This finding suggests that CYLD had no major impact on preerythrocytic parasite replication in the liver. As was seen in infections by blood transfusion, sporozoite-induced infections resulted in a significantly reduced number of parasites in *Cyld*^−/−^ mice at day 7 p.i.

### Presence of CYLD Leads to ECM-Associated Brain Pathology

**Figure 2:**
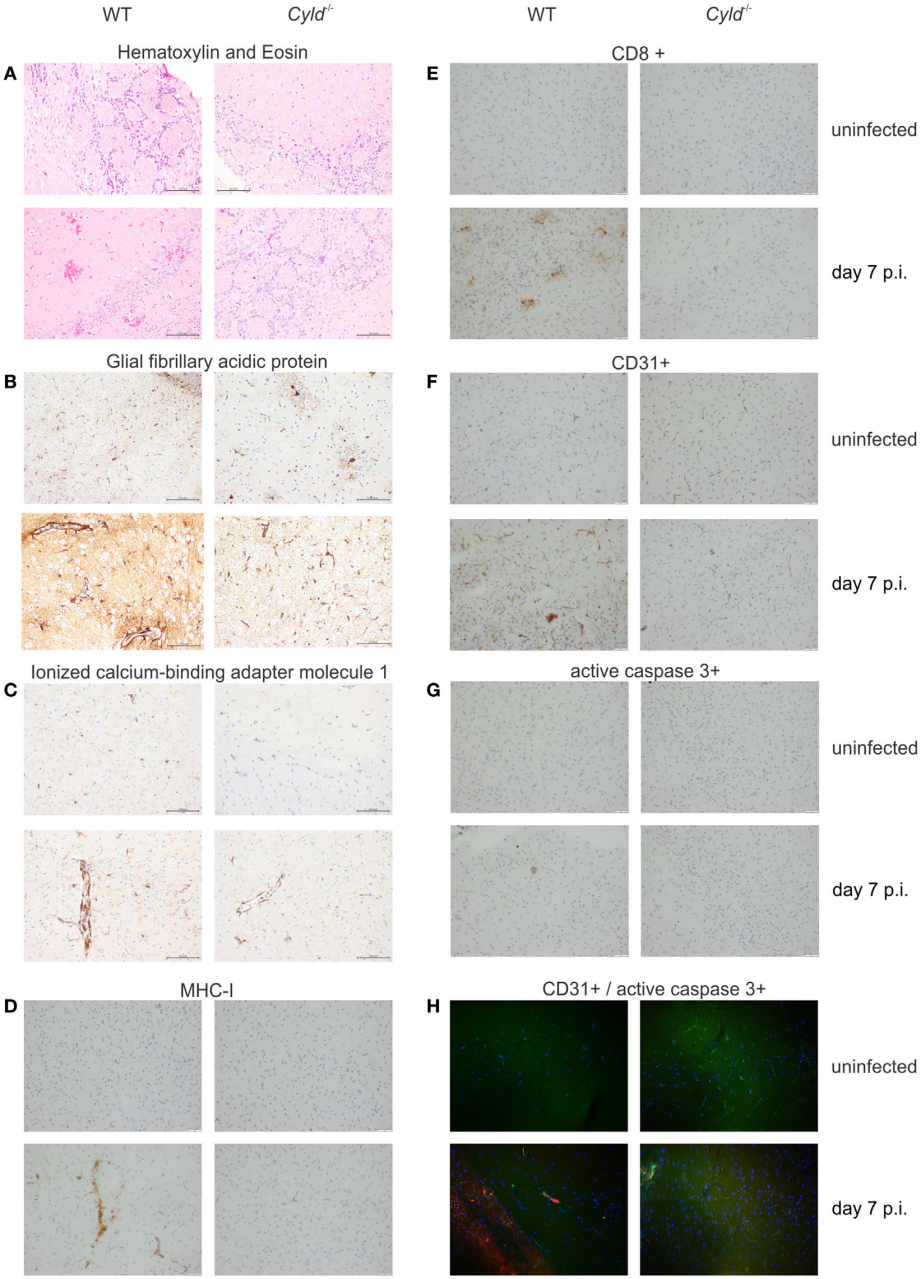
**Cylindromatosis (CYLD) augments brain pathology in experimental cerebral malaria**. Histopathology of WT and *Cyld*^−/−^ mice at day 7 postinfection (p.i.). **(A)** hematoxylin and eosin staining showing numerous hemorrhagic lesions scattered throughout the brain of WT mice at day 7 p.i. Pronounced activation of astrocytes **(B)** and microglia **(C)** in the brain of a WT mouse as shown by glial fibrillary acidic protein and ionized calcium–binding adaptor molecule 1 staining, respectively. **(D)** Strong immunostaining of MHC class I molecules on microglial cells in a brain of WT mouse. **(E)** Accumulation of CD8^+^ T cells in the brain of a WT mouse at day 7 p.i. **(F)** CD31 staining shows a prominent staining pattern on endothelial cells in the brain of a WT mouse. **(G)** Apoptosis of endothelial cells is visualized in the brain of a WT mouse by active caspase-3 staining in a vessel wall. **(H)** Double immunofluorescence shows co-localization of CD31^+^ endothelial cells (green) with active caspase 3 (red) in the brain of a WT mouse at day 7 p.i., while this is not found in a *Cyld*^−/−^ mouse. Representative staining results of three independent experiments are displayed.

To further study the effect of CYLD in ECM-associated brain pathology, a detailed histopathological analysis was performed (Figure 2). At day 7 p.i., WT mice showed severe brain hemorrhage, which was absent in *Cyld*^−/−^ mice (Figure 2A). WT, but not *Cyld*^−/−^, mice developed widespread neuroinflammation characterized by astrogliosis with increased expression of glial fibrillary acidic protein (GFAP) in activated astrocytes (Figure 2B) and ionized calcium-binding adapter molecule 1 (Iba1)^+^-ramified microglia (Figure 2C).

Furthermore, MHC class I staining on microglial cells, some of which were tightly associated with cerebral microvessels, was strongly upregulated in the brains of infected WT mice (Figure 2D). This was paralleled by focal intracerebral infiltration of CD8^+^ T cells in WT mice (Figure 2E). Interestingly, CD31/PECAM-1^+^ staining intensity on endothelial cells was considerably stronger in WT compared to *Cyld*^−/−^ mice (Figure 2F). In addition, CD31^+^ WT endothelial cells were frequently positive for cleaved caspase-3, an apoptosis-inducing effector caspase (Figures 2G,H).

Taken together, our data show that the presence of CYLD promotes brain pathology with increased neuroinflammation and hemorrhage as well as endothelial cell activation and apoptosis.

### CYLD Augments Cytokine Production in the Brain of *Pb*A-Infected Mice

**Figure 3:**
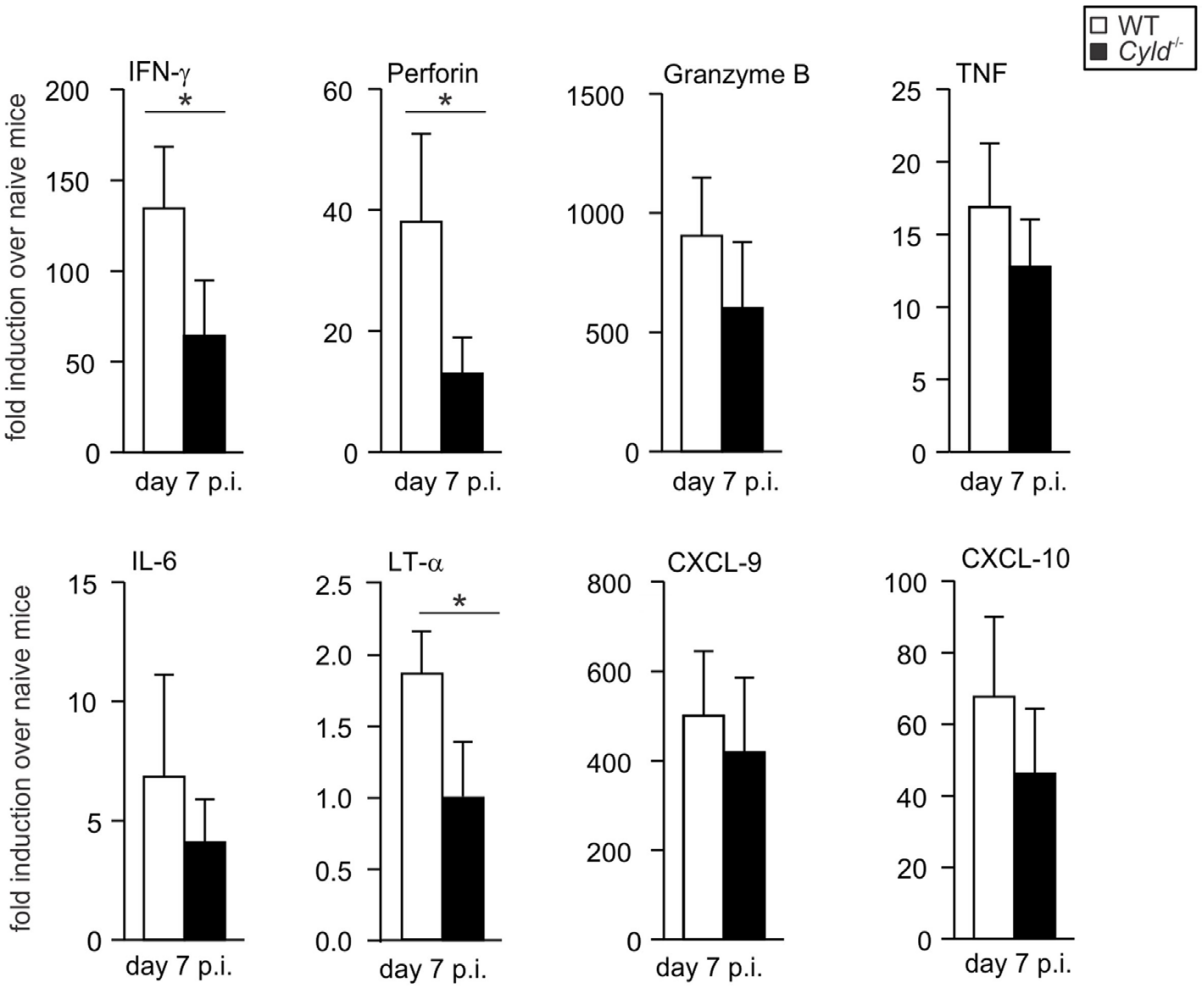
**Reduced proinflammatory cytokine expression in the brain of *Cyld*^−/−^ mice**. Quantitative reverse transcription polymerase chain reaction analysis of intracerebral cytokines *interferon-*γ, *perforin, granzyme B, tumor necrosis factor, interleukin-6, lymphotoxin-*α, and chemokines *CXCL-9* and *CXCL-10* mRNA expression in WT and *Cyld*^−/−^ mice. Data show the increase of the respective mRNA expression of *Plasmodium berghei* ANKA-infected over uninfected mice of the same mouse strain. Data represent the mean (±SD) of six mice, *p < 0.05 (two-tailed Student’s *t-*test). Data from two independent experiments are shown.

Since cytokines and chemokines regulate the immunopathological processes leading to ECM, we next determined cytokine and chemokine levels in the brain and peripheral blood of *Pb*A-infected mice. At day 7 p.i., quantitative reverse transcription polymerase chain reaction (RT-PCR) revealed that steady-state mRNA levels of *IFN-*γ, *perforin*, and *lymphotoxin* (*LT*)-α were significantly reduced in the brain of *Cyld*^−/−^ mice, while levels of *granzyme B, TNF, IL-6, CXCL-9*, and *CXCL-10* levels were comparable between the two mouse strains (Figure 3). This finding indicates that local expression of proinflammatory cytokines is significantly reduced in the absence of CYLD. This contrasts with systemic serum cytokine concentrations, since IFN-γ was increased in serum of *Cyld*^−/−^ mice as compared to WT mice, whereas the levels of IL-2, IL-4, IL-6, IL-10, IL-17, and TNF levels did not differ significantly between the two mouse strains at day 7 p.i. (Figure S1 in Supplementary Material).

### CYLD Reduces Parasite-Specific CD8^+^ T Cell Responses in Peripheral Blood

**Figure 4:**
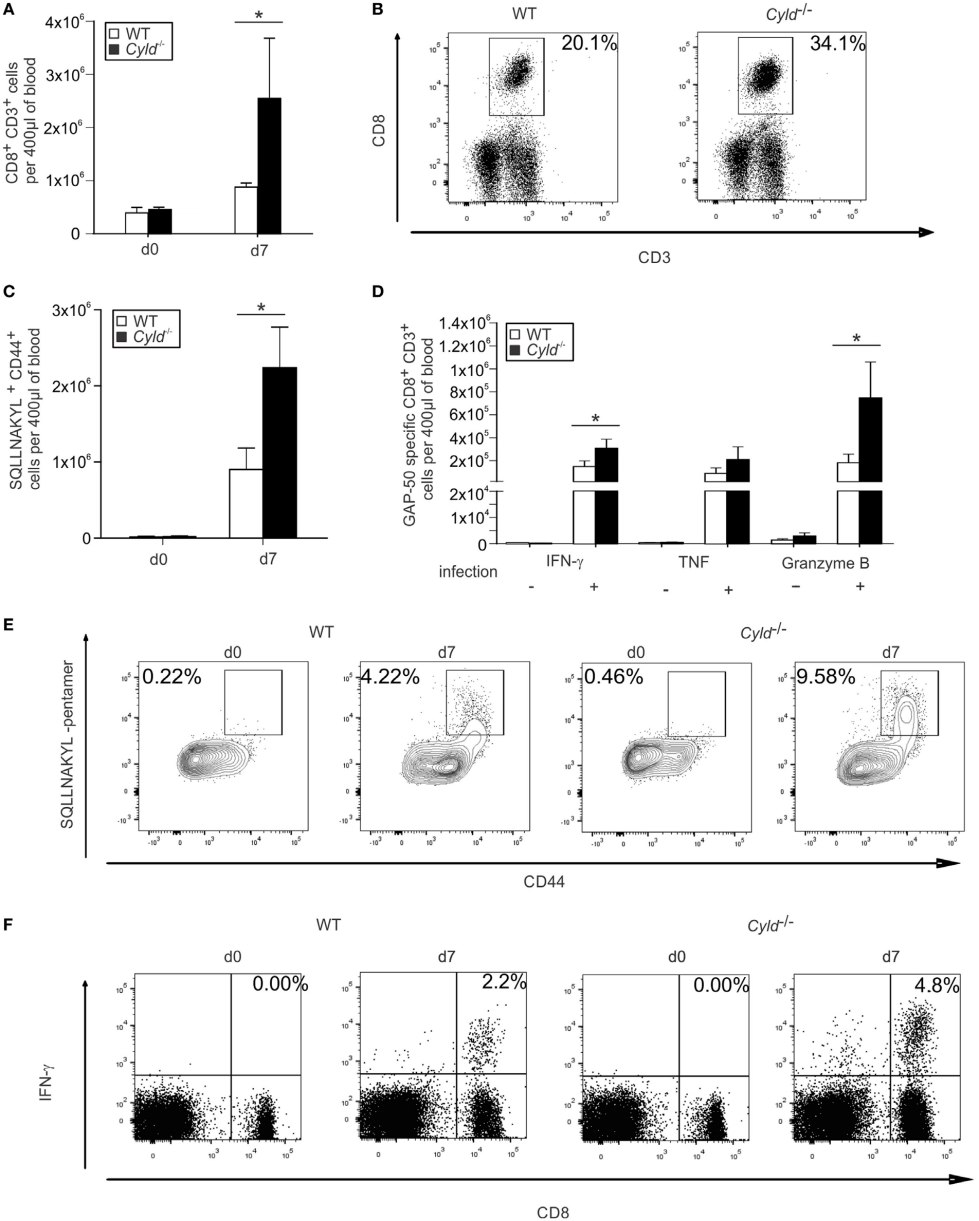
**Augmented CD8^+^ T cell responses increase in the blood of *Cyld*^−/−^ mice**. **(A,B)** Absolute **(A)** and representative relative **(B)** numbers of CD3^+^ CD8^+^ T cells of uninfected (day 0) and *Plasmodium berghei* ANKA (*Pb*A) blood stage-infected mice (day 7) were determined by flow cytometry in the blood of WT and *Cyld*^−/−^ mice (*n* = 6 each), *p < 0.05 (two-tailed Student’s *t*-test). **(C–F)** Absolute **(C)** and representative relative numbers **(E)** of H2-Db SQLLNAKYL pentamer^+^ CD44^+^ CD8^+^ T cells. Absolute **(D)** and representative relative numbers **(F)** of interferon-γ-producing CD8^+^ T cells from uninfected (day 0) and *Pb*A-infected mice at day 7 postinfection after *ex vivo* restimulation with GAP-50 peptide (*n* = 6 each), *p < 0.05 (two-tailed Student’s *t*-test). Data from one of three independent experiments are shown.

Since the parasitemia in *Cyld*^−/−^ mice was reduced and CD8^+^ T cells typically play an important role in the control of intracellular pathogens, we determined the influence of CYLD on the CD8^+^ T cell numbers in the blood during ECM. Before infection, WT and *Cyld*^−/−^ mice harbored equally low numbers of CD8*^+^* T cells (Figure 4A). On infection with *Pb*A-infected red blood cells, both the percentage and the total number of CD8^+^ T cells (Figures 4A,B), as well as *Pb*A-specific H2-Db SQLLNAKYL pentamer^+^ CD44^+^ CD8^+^ T cells, were significantly increased in *Cyld*^−/−^ mice at day 7 p.i. (Figures 4C,E).

In addition, absolute (Figure 4D) and relative (Figure 4F, Figure S2A in Supplementary Material) numbers of *Pb*A antigen-specific IFN-γ- and granzyme B-producing CD8^+^ T cells, that recognize the blood-stage *Pb*A epitope GAP-50 (36), were also significantly increased in the blood of *Cyld*^−/−^ mice. Similar results were obtained upon i.p. infection with *PbA*-infected red blood cells (Figures S2B,C in Supplementary Material). These findings indicate an association of the magnitude of antigen-specific CD8^+^ T cells with parasite clearance.

### CYLD Reduces Activation of PKC-θ and Impairs NF-κB Activation in CD8^+^ T Cells

**Figure 5:**
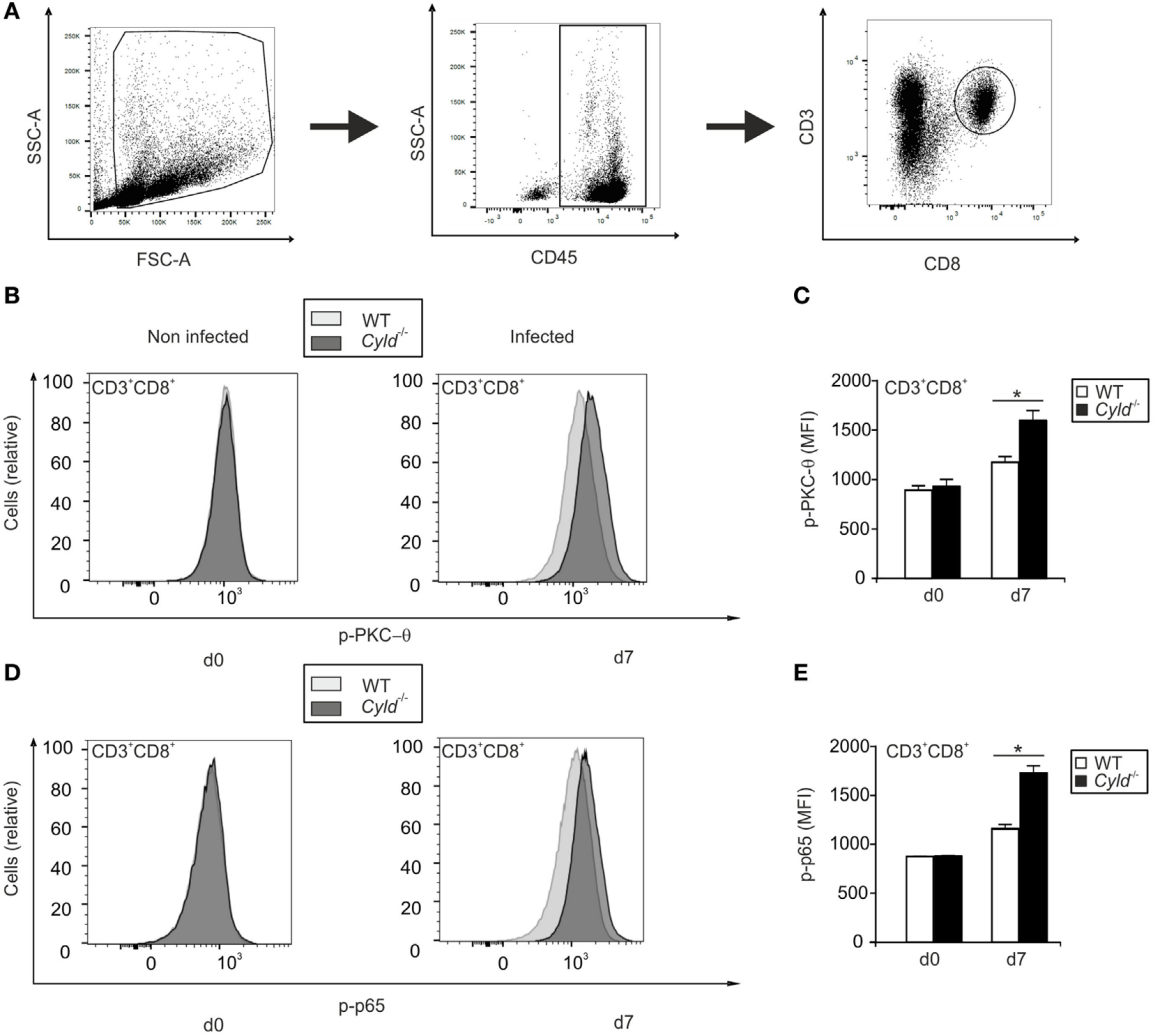
**Activation of PKC-θ and nuclear factor “kappa light-chain enhancer” of activated B cells is augmented in Cyld^−/−^ CD8^+^ T cells**. **(A)** The gating strategy used to identify CD45^+^ CD3^+^ CD8^+^ T cells is illustrated. **(B,D)** Histogram overlays show p-PKC-θ **(B)** and p-p65 **(D)** expression in CD3^+^ CD8^+^ T cells of uninfected (day 0) and infected (day 7) WT and *Cyld*^−/−^ mice. The intracellular mean fluorescence intensity of PKC-θ **(C)** and p-p65 **(E)** is shown for WT and *Cyld*^−/−^ mice at the indicated time points. Specific staining for WT mice is shown in light gray and for *Cyld*^−/−^ mice in dark gray. Data show the mean (±SD) of six mice from one of two experiments.

Since the stimulation of the T cell receptor induces NF-κB activation *via* the PKC-θ pathway (37), we performed an *ex vivo* analysis of levels of PKC-θ and p65, a constituent of the NF-κB complex, by flow cytometry in CD8^+^ T cells (Figure 5). *Pb*A infection induced increased phosphorylation of both PKC-θ and p65 in CD45^+^ CD3^+^ CD8α^+^ T cells from WT and *Cyld*^−/−^ mice (Figures 5A–E). However, phosphorylation of both PKC-θ and p65 was significantly more pronounced in *Cyld*^−/−^ mice. Thus, CYLD, which is known to interact with PKC-θ (38), impaired the activation of PKC-θ and NF-κB in CD8^+^ T cells.

### CYLD Augments CD8^+^ T Cell Response in the Brain

**Figure 6:**
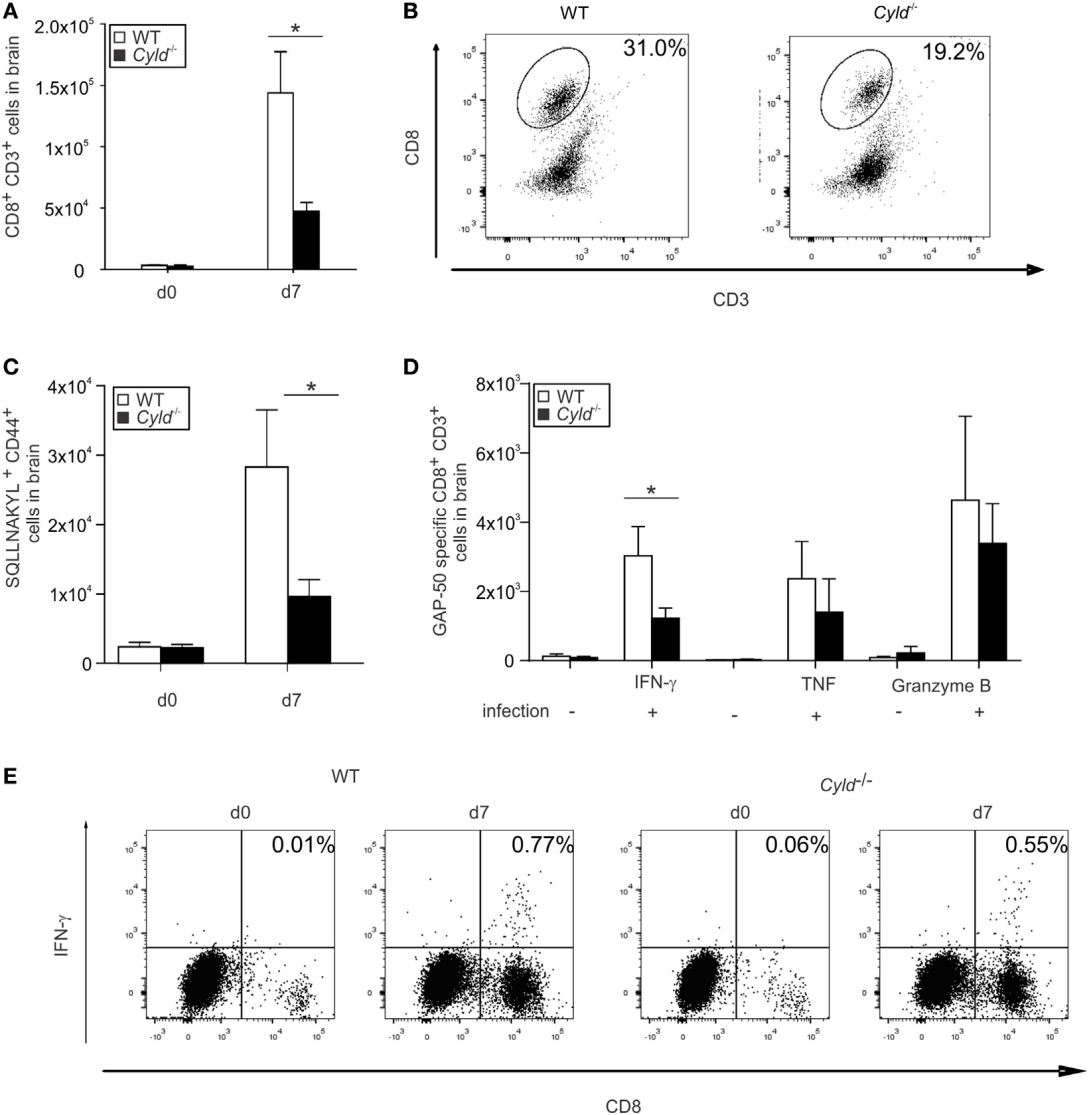
**Diminished CD8^+^ T cell response in the brain of *Cyld*^−/−^ mice**. **(A,B)** Absolute **(A)** and representative relative **(B)** numbers of CD3^+^ CD8^+^ T cells of uninfected (day 0) and *Plasmodium berghei* ANKA (*Pb*A)-blood stage-infected mice (day 7) were determined by flow cytometry in the brains of WT and *Cyld*^−/−^ mice (*n* = 6 each). **(C)** Absolute numbers of H2-Db SQLLNAKYL pentamer^+^ CD44^+^ CD8^+^ T cells. **(D)** Absolute numbers of interferon (IFN)-γ, tumor necrosis factor, and granzyme B-producing CD8^+^ T cells of uninfected (day 0) and *Pb*A-infected mice (day 7) after *ex vivo* restimulation with GAP-50 peptide. **(E)** Representative relative numbers of IFN-γ-producing CD8^+^ T cells from **(D)**, *p < 0.05 (two-tailed Student’s *t*-test). **(A–E)** Data from one of three independent experiments are shown.

Since pathogenic CD8^+^ T cells mediate end-stage immune pathology in ECM [reviewed in Ref. (39)], the intracerebral CD8^+^ T cell response of WT and *Cyld*^−/−^ mice were analyzed in detail by flow cytometry (Figure 6). Uninfected WT and *Cyld*^−/−^ mice harbored very low numbers of CD8^+^ T cells in the brain (Figure 6A). Upon infection, both the percentage and the total number of CD8^+^ T cells (Figures 6A,B), as well as *Pb*A-specific H2-Db SQLLNAKYL pentamer^+^ CD44^+^ CD8^+^ T cells (Figure 6C), were significantly increased in the brains of WT mice at day 7 p.i. Numbers of *Pb*A GAP-50-specific CD8^+^ T cells producing IFN-γ, TNF, and granzyme B were reduced in the brains of *Cyld*^−/−^ mice (Figure 6D). However, only differences for IFN-γ were statistically significant upon infection with blood-stage *Pb*A (Figures 6D,E). We could independently confirm these data by sporozoite infection (Figure S3 in Supplementary Material). Together, our findings corroborate the notion that antigen-specific CD8^+^ T cells might contribute to ECM pathology.

### CYLD Impairs Parasite Clearance by CD8^+^ T Cells

**Figure 7:**
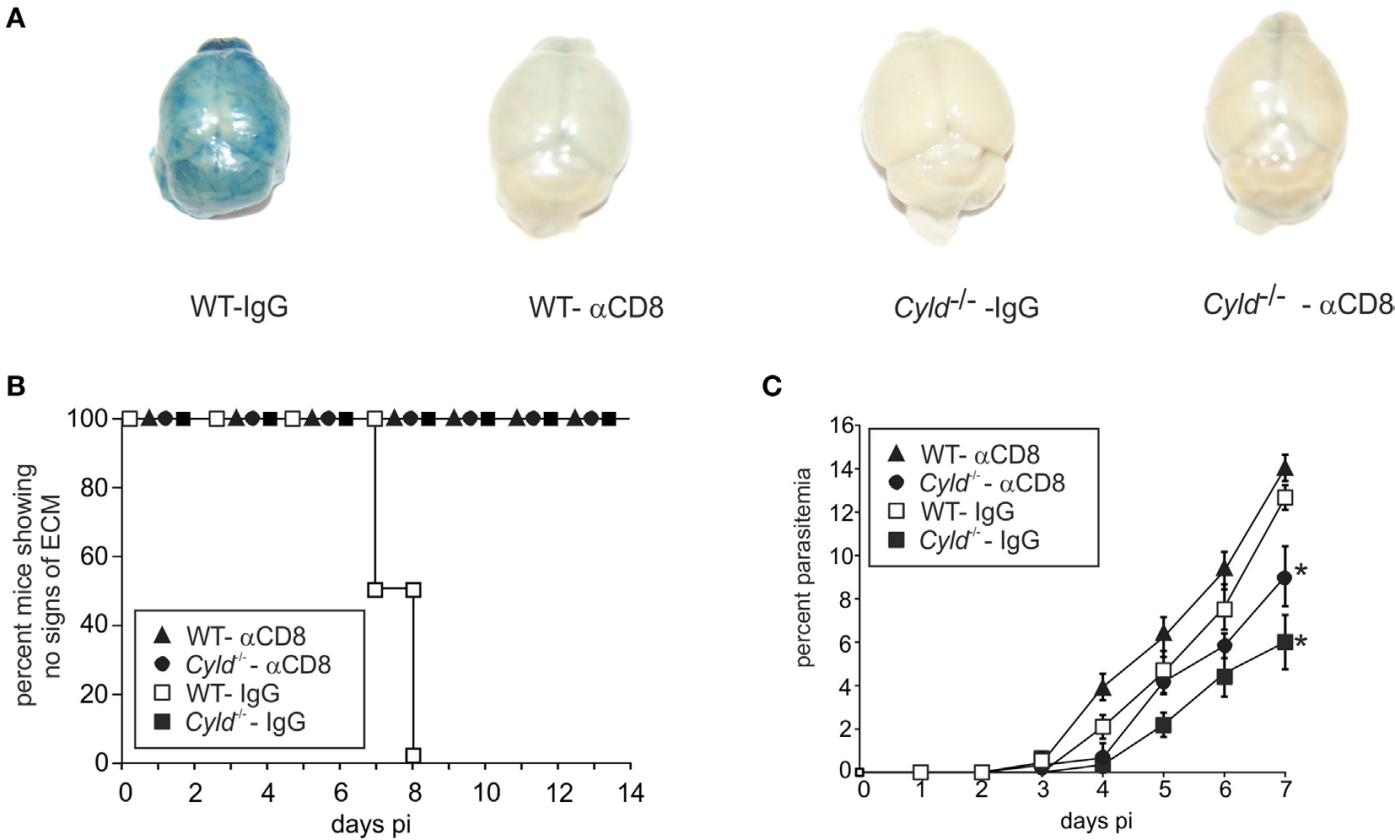
**Depletion of CD8^+^ T cells rescues mice from experimental cerebral malaria**. C57BL/6 *Cyld*^−/−^ and WT (*n* = 6 each) mice were either treated with anti-CD8 antibody or rat IgG starting 3 days before intraperitoneal (i.p.) infection and i.p. 1 × 10^6^ *Plasmodium berghei* ANKA (*Pb*A)-infected red blood cells. **(A)** At day 7 postinfection (p.i.), Evans Blue was injected intravenously in mice. The mice were euthanized 1 h later and perfused with saline, and the isolated brains were photographed. Representative images from two independent experiments are shown. **(B)** Survival was monitored daily until day 14 p.i. **(C)** The percentage of parasitized erythrocytes in the peripheral blood was enumerated daily from Giemsa-stained thin blood smears. **(A–C)** *p < 0.05 (two-tailed Student’s *t*-test) Representative data from two independent experiments are shown.

To determine whether the improved parasite control of *Cyld*^−/−^ mice is mediated by CYLD*-*deficient CD8^+^ T cells, we depleted CD8^+^ T cells in WT and *Cyld*^−/−^ mice before infection (Figure S4 in Supplementary Material) and assessed the survival, vascular leakage, and parasite load. Infected WT and *Cyld*^−/−^ mice with CD8^+^ T cell depletion showed no vascular leakage (Figure 7A) and survived the infection (Figure 7B), but harbored increased parasite loads compared to the WT and *Cyld*^−/−^ mice receiving control antibody (Figure 7C). However, the parasite load in the CD8^+^ T cell-depleted *Cyld*^−/−^ mice was always lower compared to CD8^+^ T cell-depleted WT mice, indicating that in addition to the contribution of CD8^+^ T cells the enhanced parasite control in the *Cyld*^−/−^ mice is also mediated by other cell types, likely *Cyld*^−/−^ macrophages and granulocytes.

### CYLD Augments Cytokine Production but Impairs Chemokine Receptor Expression by CD8^+^ T Cells

**Figure 8:**
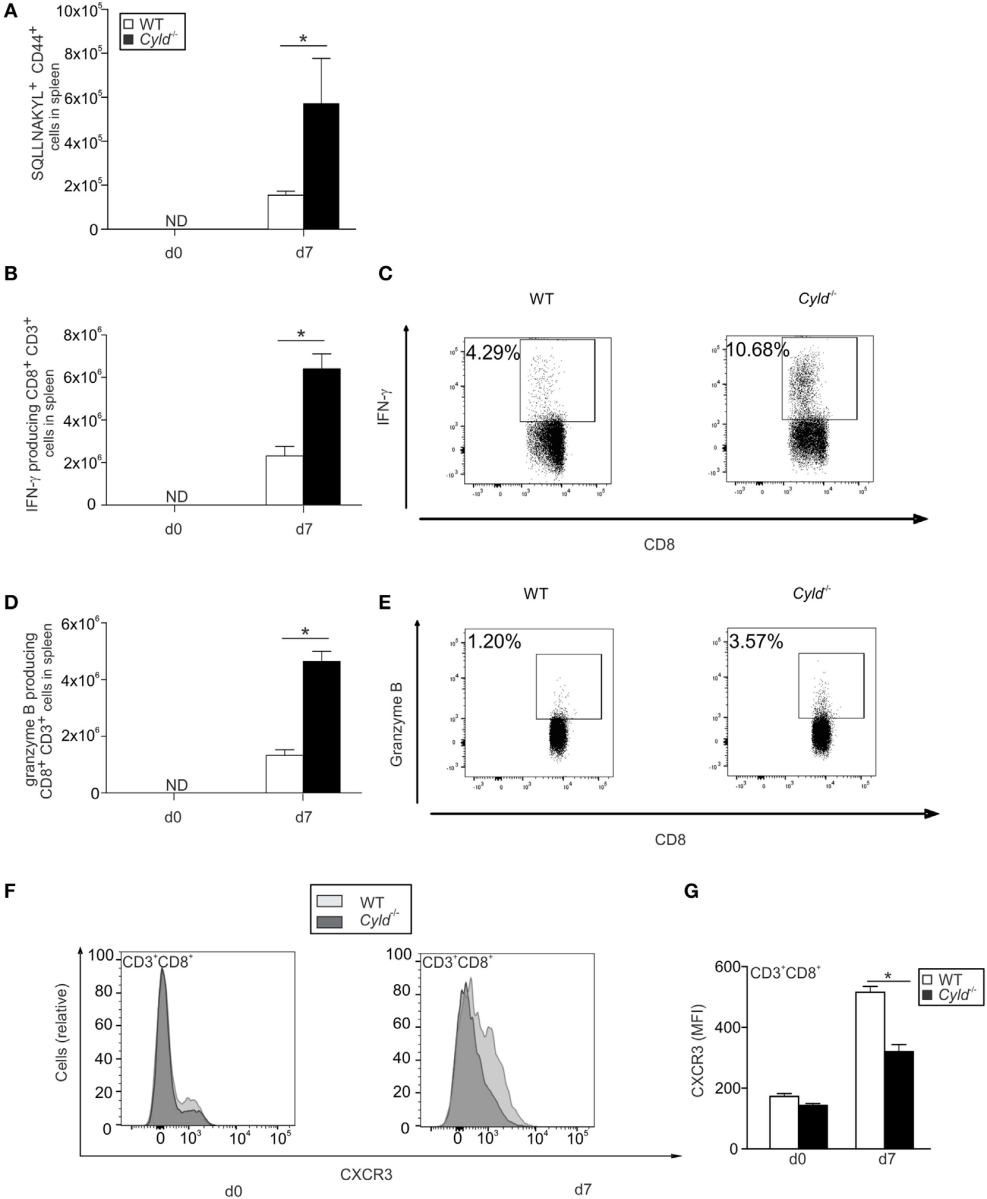
**Enhanced CD8^+^ T cell responses but reduced CXCR3 expression by CD8^+^ T cells in the spleen of *Cyld*^−/−^ mice**. **(A)** Absolute numbers of SQLLNAKYL pentamer^+^ CD44^+^ CD8^+^ T cells of uninfected mice (day 0) and mice infected with *Plasmodium berghei* ANKA (*Pb*A)-blood stage parasites (1 × 10^6^ intraperitoneally) were determined by flow cytometry in the brains of WT and *Cyld*^−/−^ mice at day 7 postinfection (p.i.) (*n* = 6 each). **(B–E)** Absolute **(B)** and relative **(C)** numbers of interferon-γ-producing CD8^+^ T cells and absolute **(D)** and relative **(E)** numbers granzyme B-producing CD8^+^ T cells from uninfected mice (day 0) and *Pb*A-infected mice at day 7 p.i. after *ex vivo* restimulation with GAP-50 peptide (*n* = 6 each), *p < 0.05 (two-tailed Student’s *t*-test). Data from one of three independent experiments are shown. **(F)** Histogram overlays show data for CXCR3 expression in CD3^+^ CD8^+^ T cells of uninfected (day 0) and infected (day 7) WT and *Cyld*^−/−^ mice. **(G)** The intracellular mean fluorescence intensity of CXCR3 is shown for WT and *Cyld*^−/−^ mice at the indicated time points. **(A,C,E)** ND = non-detectable. **(A,C,E)** Data show the mean (±SD) of six mice from one of two experiments.

Although the parasite-specific CD8^+^ T cell response was enhanced in the peripheral blood of *Cyld*^−/−^ mice, these mice harbored reduced numbers of pathogen-specific CD8^+^ T cells in the brain. Since chemokine receptors are essential for migration of CD8^+^ T cells, we checked for the cell numbers, cytokine production and the chemokine receptor expression of parasite-specific CD8^+^ T cells in the spleen. Upon infection with blood-stage *Pb*A, the total number of parasite-specific CD8^+^ T cells were strongly increased in the spleen of *Cyld*^−/−^ mice at day 7 p.i. (Figure 8A).

In addition, absolute (Figures 8B,D) and relative (Figures 8C,E) numbers of *Pb*A antigen-specific IFN-γ- and granzyme B-producing GAP-50-specific CD8^+^ T cells were significantly increased in the spleen of infected *Cyld*^−/−^ mice. In marked contrast, expression of the chemokine receptor CXCR3 was significantly reduced in *Cyld*^−/−^ CD8^+^ T cells (Figures 8F,G). A small, albeit non-significant, reduction in *Cyld*^−/−^ CD8^+^ T cells was also detected for CCR5 (Figures S5A,B in Supplementary Material) and CCR1 (Figures S5C,D in Supplementary Material). Since CXCR3 plays an important role in migration of CD8^+^ T cells to the brain (40), the reduced accumulation of CD8^+^ T in the brain of *Cyld*^−/−^ mice may be attributed to reduced levels of CXCR3.

### CD4^+^ T Cell Responses Are Augmented in the Blood but Reduced in the Brain of *Cyld*^−*/*−^ Mice

**Figure 9:**
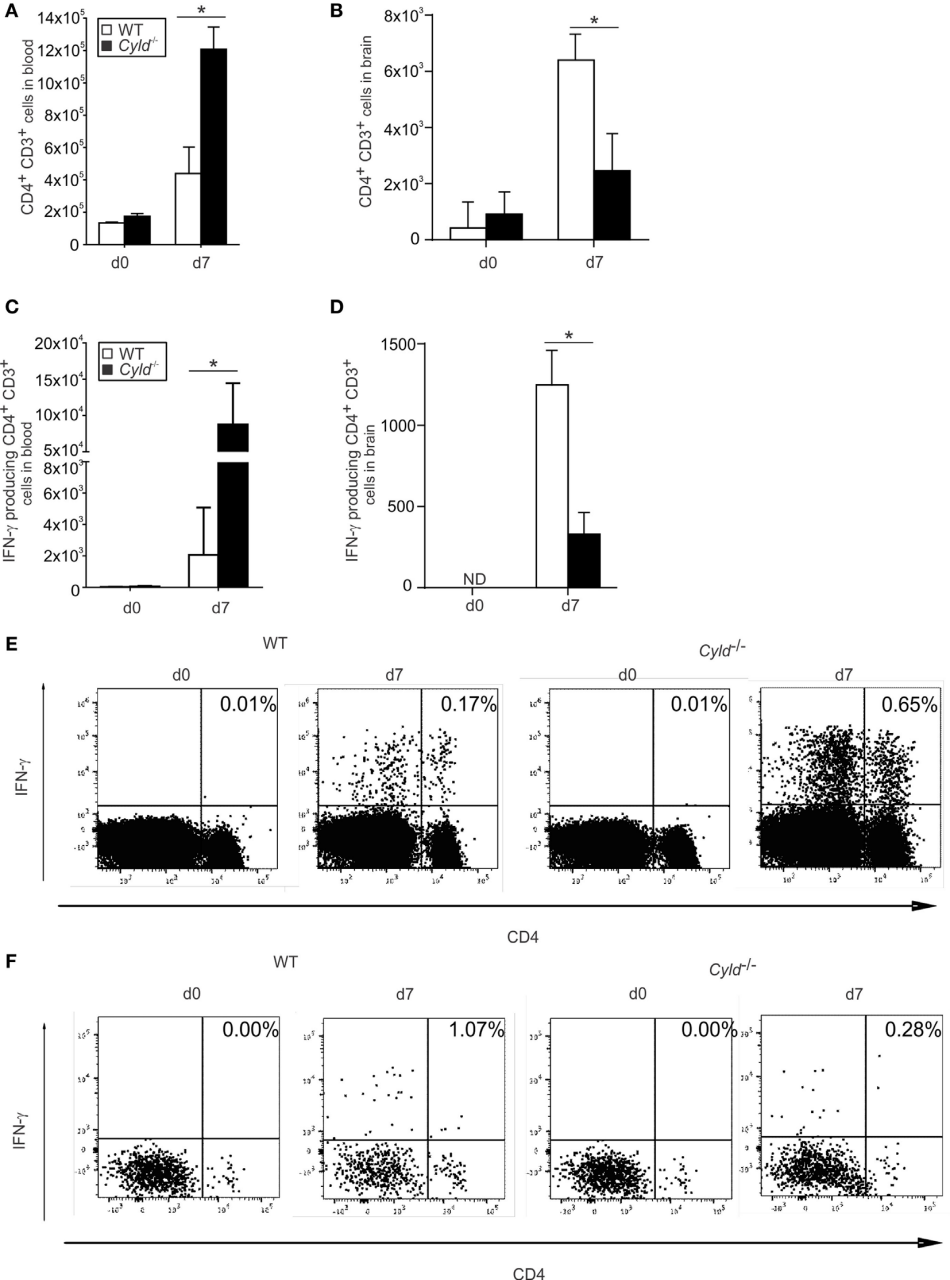
**CD4^+^ T cell responses increase in the blood and decrease in the brain of *Cyld*^−/−^ mice**. **(A,B)** Total numbers of CD3^+^ CD4^+^ T cells in blood **(A)** and brain **(B)** of uninfected (day 0) and *Plasmodium berghei* ANKA (*Pb*A)-infected (day 7) WT and *Cyld*^−/−^ mice were determined by flow cytometry (*n* = 6 each), *p < 0.05 (two-tailed Student’s *t*-test) **(C–F)** Absolute **(C)** and representative relative **(E)** numbers of interferon (IFN)-γ-producing CD4^+^ T cells in blood and absolute **(D)** and representative relative **(F)** numbers of IFN-γ-producing CD4^+^ T cells in the brain from uninfected (day 0) and *Pb*A-infected mice (day 7) after *ex vivo* restimulation with anti-CD3/CD28 (*n* = 6 each), *p < 0.05 (two-tailed Student’s *t*-test). Data from one of three independent experiments are shown.

Since CD4^+^ T cells also play an important role in the control of blood-stage malaria, we determined the influence of CYLD on the CD4^+^ T cell numbers in the blood and brain during ECM. Before infection, WT and *Cyld*^−/−^ mice harbored equal numbers of CD4*^+^* T cells in the blood (Figure 9A) and brain (Figure 9B). Upon infection with *PbA*-infected red blood cells, the total number of CD4^+^ T cells increased in the blood and brain of both mouse strains. However, the numbers of CD4^+^ T cells were strongly increased in the blood (Figure 9A), but markedly reduced in the brain of *Cyld*^−/−^ mice (Figure 9B).

In addition, absolute (Figure 9C) and relative (Figure 9E) numbers of *Pb*A antigen-specific IFN-γ-producing CD4^+^ T cells were significantly increased in the blood of *Cyld*^−/−^ mice, but lower in the brain of *Cyld*^−/−^ mice (Figures 9D,F). These findings indicate that similar to CD8*^+^* T cells, the CD4*^+^* T cell response to *PbA* is also regulated by CYLD.

### Absence of ECM in Infected *Cyld*^−*/*−^ Mice Is Mediated by Both Hematopoietic and Parenchymal Cells

**Figure 10:**
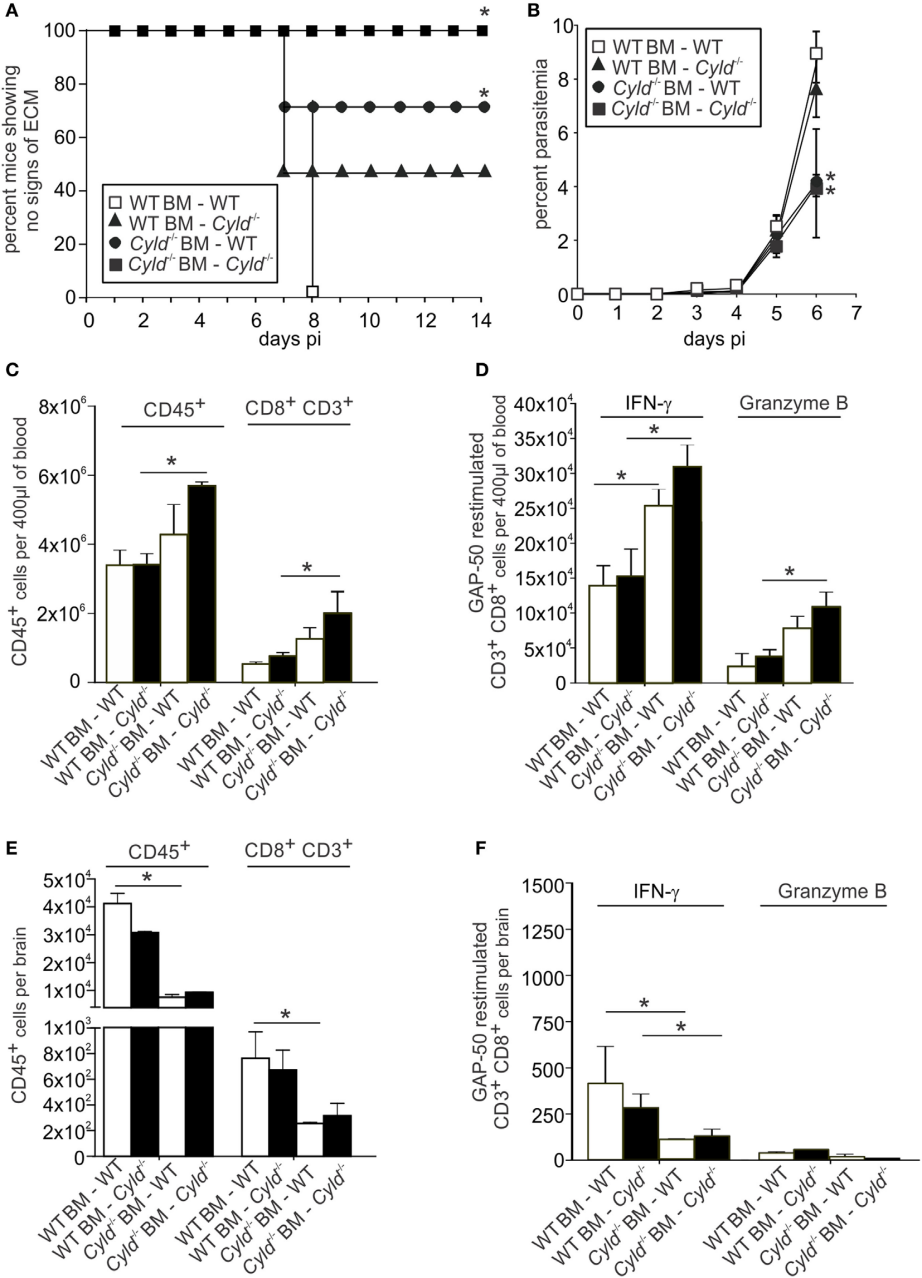
**Deletion of *Cyld* in both the hematopoietic and the parenchymal compartment contributes to protection from experimental cerebral malaria**. **(A–F)** A total of 10 × 10^6^ Bone marrow cells isolated from WT and *Cyld*^−/−^ mice, respectively, and 10 × 10^6^ cells were adoptively transferred into sublethally irradiated (one dose of 12 Gy) WT and *Cyld*^−/−^ mice as indicated. Eight weeks after bone marrow transfer, chimeras were infected with 1 × 10^6^ *Plasmodium berghei* ANKA (*Pb*A) parasitized red blood cells. **(A)** The survival of mice was monitored until day 14 postinfection. **(B)** The percentage of parasitized erythrocytes in the peripheral blood was enumerated daily from Giemsa-stained thin blood smears. Data represent the mean ± SD (*n* = 4 each). **(C,E)** Absolute numbers of total CD45^+^ leukocytes in the blood **(C)** and brain **(E)** of *Pb*A-infected mice (day 7). **(D,F)** Absolute number of interferon-γ- and granzyme B-producing CD8^+^ T cells from blood **(D)** and brain **(F)** of *Pb*A-infected mice (day 7) after *ex vivo* restimulation with GAP-50 peptide. **(A–F)** *p < 0.05 (two-tailed Student’s *t*-test). Representative data from one of two experiments are shown.

To validate whether the lethal course of cerebral malaria in the WT mice was caused by hematopoietic or parenchymal cells, reciprocal bone marrow chimera of WT and *Cyld*^−/−^ mice were generated (Figure 10). Eight weeks after bone marrow transplantation, mice were infected with *Pb*A-parasitized red blood cells. Seventy-five percent of WT mice reconstituted with *Cyld*^−/−^ bone marrow did not develop clinical signs of ECM, whereas 50% of *Cyld*^−/−^ mice reconstituted with WT bone marrow did show signature symptoms of ECM (Figure 10A). In accordance with non-irradiated mice, all *Cyld*^−/−^ mice reconstituted with *Cyld*^−/−^ bone marrow remained healthy, whereas all WT mice reconstituted with WT bone marrow developed signature ECM symptoms. Thus, the clinical consequences of infection appear to depend on both *Cyld*-expressing hematopoietic and parenchymal cells from donor and recipient animals, respectively.

Furthermore, WT mice reconstituted with *Cyld*^−/−^ bone marrow harbored low parasite numbers in the blood. Lower parasitemia was comparable with the *Cyld*^−/−^ mice reconstituted with *Cyld*^−/−^ bone marrow (Figure 10B). In contrast, *Cyld*^−/−^ mice reconstituted with WT bone marrow harbored significantly higher parasite burden, which in turn was comparable to WT mice reconstituted with WT bone marrow (Figure 10B). These data indicate an exclusive contribution of hematopoietic cells in the parasite control during infection.

To further analyze whether transplantation of *Cyld*^−/−^ bone marrow cells into WT mice improved parasite-specific T cell responses in WT mice, the number of GAP-50-specific CD8^+^ T cells was determined. In blood, the total number of CD8^+^ T cells (Figure 10C) as well as IFN-γ- and granzyme B-producing GAP-50-specific CD8^+^ T cells (Figure 10D) was increased in WT mice receiving *Cyld*^−/−^ bone marrow, suggesting an important contribution of CYLD in the hematopoietic cell population from the donor mice in modulating systemic CD8^+^ T cell responses.

In the brains of bone marrow chimeric mice, significantly reduced numbers of the total CD8^+^ T cells (Figure 10E), as well as IFN-γ and granzyme B-producing GAP-50-specific CD8^+^ T cells (Figure 10F), were detected in WT mice receiving *Cyld*^−/−^ bone marrow. Strikingly, the reverse transfer, i.e., WT bone marrow into *Cyld*^−/−^ mice, resulted in similar low numbers of total and antigen-specific T cells. These results demonstrate that the observed lack of ECM symptoms in *Cyld*^−/−^ mice can be attributed to both hematopoietic and parenchymal cells.

## Discussion

This study identifies for the first time a DUB as a crucial host factor contributing to the lethal course of ECM. Our detailed comparative analysis of *P. berghei* infection, and the corresponding host immune responses in normal and *Cyld*^−/−^ mice showed that the presence of CYLD impaired parasite control and augmented brain pathology by promoting hemorrhage, neuroinflammation with activation of astrocytes and microglia, increased accumulation of CD8^+^ T cells, and apoptosis of endothelial cells. During the pre-erythrocytic stage of the infection, the parasite liver stages replicate in host hepatocytes for 2–3 days before they enter the blood stream. Our study shows that upon both sporozoite and blood-stage infection, parasitemia was detectable in WT and *Cyld*^−/−^ mice on day 3 p.i., indicating that *CYLD* deficiency did not prevent parasite replication in the liver. In this study, we addressed the role of CYLD in primary *Plasmodium* infections and can exclude a critical role in pre-erythrocytic parasite development and life cycle progression to blood infection, the only parasite stage that causes malaria. Future work is warranted to study a potential influence of CYLD on the hepatic immune response and acquisition of protective immunity after multiple sporozoite immunizations.

In marked contrast, the numbers of infected erythrocytes were significantly reduced in *Cyld*^−/−^ mice upon both sporozoite or blood-stage *Pb*A-induced infection. This is in contrast to our observation in listeriosis (11, 12). *Listeria monocytogenes* (Lm) also replicates in the hepatocytes and additionally in the macrophages. We could show previously that CYLD inhibited protective hepatocytic and macrophage responses and impaired the control of Lm (11, 12).

In both sporozoite and asexual blood stage infections, the systemic CD8^+^ T-cell response was significantly augmented when CYLD was absent. Previous studies have consistently shown that CD8^+^ T cells play no role in protection against blood-stage *PbA* infection (41–44). More recent studies have challenged this view by showing a major role for parasite-specific CD8^+^ T cells in acute and chronic blood-stage infection (45). In this study, we demonstrated a strikingly enhanced CD8^+^ T cell response following acute blood-stage *PbA* infection in mice that lack the central regulator *CYLD*.

PKC-θ is a key component in the activation of T cells. Our previous studies have shown that mice deficient in PKC-θ exhibit impaired T cell activation and protection against infectious diseases due to reduced activation of NF-κB pathway (46, 47). Thuille et al. (38) demonstrated that PKC-θ interacts with CYLD leading to inhibition of CYLD function (38). Interestingly, we observed increased activation of PKC-θ and NF-κB in CYLD-deficient CD8^+^ T cells, suggesting that the interaction between CYLD and PKC-θ might also lead to the inhibition of PKC-θ-mediated NF-κB activation.

*Plasmodium berghei* ANKA infection was associated with an increased expansion of pathogen–specific CD8^+^ T cells in *Cyld*^−/−^ mice, which in turn was accompanied by augmented production of IFN-γ, TNF, granzyme B, and perforin. These cytokines are known to contribute to the control of *Pb*A blood parasites. Although our CD8^+^ T cell depletion experiments show the importance of CD8^+^ T cells in the control of *Pb*A blood parasites, CD8^+^ T cell depleted *Cyld*^−/−^ mice harbored lower parasite loads compared to CD8^+^ T cell-depleted WT mice, indicating that the enhanced parasite control in the *Cyld*^−/−^ mice is also mediated by other immune cells in addition to CD8^+^ T cells.

We have shown before that IFN-γ-stimulated *Cyld*^−/−^ macrophages have an enhanced capacity to control intracellular pathogens (12). Furthermore, it is known that IFN-γ-stimulated macrophages exhibit enhanced phagocytosis of parasitized red blood cells (48). Our observation of reduced pathogen load in the blood of *Cyld*^−/−^ mice at day 5 p.i., i.e., before the onset of clinical symptoms of ECM, indicates a possible role of macrophages in the enhanced clearance of parasitized red blood cells in the *Cyld*^−/−^ mice.

Although CYLD is an important inhibitor of proinflammatory signaling pathways and reduces production of cytokines and chemokines, intracerebral cytokine and chemokine production of *Pb*A-infected *Cyld*^−/−^ mice was reduced compared to WT mice. Reduced accumulation of pathogen-specific cytokine-producing CD8^+^ T cells could be attributed to reduced expression of the chemokine receptor CXCR3 on the *Cyld*^−/−^ CD8^+^ T cells, since CXCR3 expression on CD8^+^ T cells is required for T cell migration into the brain (40) and the development of ECM. Currently, it is unclear how CYLD regulates CXCR3 expression, and to clarify this aspect, further studies are required. One interpretation of this spatial reallocation of CD8^+^ T cells might be that an increased parasite load is a critical factor leading to intracellular cytokine and chemokine production and intracerebral accumulation of CD8^+^ T cells. In good agreement with the ameliorated course of ECM and the reduced production of intracellular proinflammatory cytokines, brain pathology including blood–brain barrier breakdown, perivascular bleeding, activation of astrocytes, and microglia were reduced in the *Cyld*^−/−^ mice.

CD4^+^ T cells play an important role in controlling blood-stage malaria (49). Our observation of an enhanced CD4^+^ T cell response in blood of *Cyld*^−/−^ mice indicates that CYLD also impaired CD4^+^ T cell-mediated protection against *Pb*A infection. We also report that the expression of active caspase 3, the effector caspase-inducing apoptosis of endothelial cells, was increased in *Cyld*-expressing WT mice. Since *Cyld*^−/−^ mice are protected from apoptosis due to increased NF-κB activation (7), this mechanism may also play an important protective role in *Pb*A-infected *Cyld*^−/−^ mice, and future studies on pathogen-induced apoptosis of endothelial cells are warranted.

In support of a role of CYLD in parenchymal, and, perhaps, endothelial, cells, our bone marrow chimera experiments demonstrate that *Cyld* expression in radioresistant parenchymal cells contributed to the development of lethal ECM. However, complete protection from death was dependent on *Cyld* deficiency in donor and recipient mice illustrating that CYLD inhibited protective host responses both in the immune system and in parenchymal cells.

Currently, inhibitors of CYLD and other DUBs are under clinical development, since DUBs are attractive candidate molecules in different diseases, including cancer (50). Our data indicate that CYLD inhibition might also be an attractive therapeutic option in severe malaria in combination with antiparasitic drugs.

## Materials and Methods

### Ethics Statement

All animal experiments were in compliance with the German Animal Welfare Act (TierSchG) in a protocol approved by the Landesverwaltungsamt Sachsen-Anhalt (file number: 203.h-42502-2-901, University of Magdeburg).

### Animals

Age- and sex-matched animals were used for the experiments. C57BL/6 WT were obtained from Janvier (Le Genest Saint Isle, France), and C57BL/6 *Cyld*^−/−^ mice were kindly provided by Dr. Ramin Massoumi (Department of Laboratory Medicine, Malmö, Sweden) (9). The C57BL/6 *Cyld*^−/−^ mice were backcrossed with C57BL/6 mice for 10 generations. All animals were kept under conventional conditions in an isolation facility of the Otto-von-Guericke University Magdeburg. Experiments were approved and supervised by local governmental institutions.

### Parasite Infection

*Plasmodium berghei* strain ANKA was used for the experiments. For the hepatic stage infection, mice were infected i.v. with 20,000 live sporozoites obtained from salivary gland homogenate of day 21 *Pb*A-infected female *Anopheles* mosquitoes. For blood-stage infection, parasites were passaged in C57BL/6J mice, and stabilates were harvested and stored in liquid nitrogen (100 µl of 1 × 10^6^ infected red blood cells in 200 µl Alsever’s solution and 10% glycerol). For infection, 1 × 10^6^ *Pb*A-infected red blood cells were injected i.p., with the dose adjusted for each stabilate batch such that neurological signs manifest 7 days later in the majority of mice. The infected mice were monitored for neurological symptoms, i.e., paralysis, ataxia, convulsions, and coma occurring between day 6 and 10 p.i. (51). Parasitemia was measured daily from day 3 p.i. onward by microscopic examination of Giemsa-stained thin blood smears.

### Evans Blue Staining

Mice were i.v. injected with 100 µl of a 1% solution of Evans blue in phosphate-buffered saline (PBS) at day 7 p.i. One hour later, the mice were perfused intracardially with 0.9% NaCl after isoflurane anesthesia. Brains were removed and photographed.

### Histopathology

For immunohistochemistry and immunofluorescence on frozen sections, mice were perfused intracardially with 0.9% NaCl after isoflurane anesthesia. For histology on paraffin sections, anesthetized mice were perfused with 4% paraformaldehyde in PBS and brain was removed and fixed with 4% paraformaldehyde for 24 h. Paraffin sections (4 µm) were used for hematoxylin and eosin staining and immunostaining with antibodies against GFAP and Iba1. A total of 7 µm cryostat sections were used for immunostains with active caspase-3 and CD31. Immunostaining for these two fluorescent markers was performed in staining chambers after fixation in acetone and chloroform for 10 and 8 min, respectively. The sections were then blocked with appropriate sera (1:10 in PBS) dependent on the source of the secondary antibody and incubated with the aforementioned primary antibodies at room temperature. After washing, the secondary antibody was added for 1 h. For this double immunostaining, the protocol was performed using the first primary antibody, and afterward, the same protocol was repeated with the second primary antibody and appropriate secondary antibodies. After a final washing step, the sections were aqueously mounted and stored at 4°C. Images were acquired with an Olympus microscope BX50 and the digital camera DP25 as well as cell Sens software (all by Olympus, Hamburg, Germany).

### RT-PCR

Isolation of mRNA from the brains of uninfected and *Pb*A-infected mice was performed with an RNAeasy kit (Qiagen, Hilden, Germany). The SuperScript reverse transcriptase kit with oligo (dT) primers (Invitrogen) was used to transcribe mRNA into cDNA. Quantitative RT-PCR for *IFN-*γ, *perforin, granzyme B, TNF, IL-6, CXCL-9, CXCL-10, LT-*α, and *hypoxanthine phosphoribosyltransferase* (*HPRT*) was performed with cDNA from C57BL/6 WT and C57BL/6 *Cyld*^−/−^ mice and the respective Taqman gene expression assay (Applied Biosystems, Darmstadt, Germany). Amplification was performed with a GeneAmp 5700 sequence detection system (Applied Biosystems). Quantitation was performed with the sequence detector software SDS (version 2.1; Applied Biosystems), according to the ΔΔ*C_T_* threshold cycle method with HPRT as the housekeeping gene (52). Data are expressed as the increase in the level of mRNA expression in infected mice over that in uninfected controls of the respective mouse strain. All primers and probes were obtained from Applied Biosystems.

### Leukocyte Isolation

Before isolation of cerebral leukocytes, the animals were anesthetized with isoflurane (Baxter, Deerfield, IL, USA) and intracardially perfused with 0.9% NaCl to remove contaminating intravascular leukocytes from the brain. Thereafter, brain tissue was minced through a 100-μm pore size cell strainer, and leukocytes were separated by Percoll (GE Healthcare, Freiburg, Germany) density gradient centrifugation. Peripheral blood was obtained by cardiac puncture. The erythrocytes were lysed with ammonium chloride, washed twice with ice-cold PBS at 300 × *g* for 10 min, and the leukocyte numbers were counted.

### Flow Cytometry

Leukocytes isolated from the blood and brain were analyzed by flow cytometry on a FACS Canto II with Cell Quest software (both from BD Biosciences, Heidelberg, Germany). Cells were stained with anti-CD45 in combination with anti-CD8 and anti-CD3 for CD8^+^ T cells. All antibodies were obtained from BD Biosciences. For intracellular staining of cytokines, splenocytes were stimulated with GAP-50 peptide (SQLLNAKYL, 10 µg/ml) (36) in RPMI-1640 at 37°C for 4 h according to cytokine stimulation protocols by BD. After stimulation, cells were stained for CD8^+^ T cells as described earlier. Thereafter, cells were fixed with Cytofix/Cytoperm, permeabilized with Perm Wash (BD Biosciences) and stained with intracellular antibodies against IFN-γ, TNF, and granzyme B.

### Generation of Bone Marrow Chimera

Bone marrow was collected from WT and *Cyld*^−/−^ mice. Femur and tibia were aseptically removed from mice, the bone ends were cut off, and the bone marrow cells were flushed out using PBS (4–8°C; Gibco, Darmstadt, Germany). For each chimera, 10 × 10^6^ cells of bone marrow cells were transferred i.v. into lethally irradiated (one dose of 12 Gy) WT or *Cyld*^−/−^ recipients. Recipient mice were allowed 8 weeks for reconstitution before infection with 1 × 10^6^ *Pb*A-infected red blood cells.

### Cytometric Bead Array

The serum levels of IL-2, IL-4, IL-6, IL-10, IL-17, IFN-γ, and TNF of WT and *Cyld*^−/−^ mice were analyzed by flow cytometry using the Cytometric Bead Array from BD Biosciences using the manufacturer’s protocol. Peripheral blood obtained by cardiac puncture was centrifuged (490 × *g*, 5 min) to harvest the serum. Cytokine concentration in the serum was determined by adding 50 µl of cytokine-specific capture bead mixture to 50 µl of supernatant. Phycoerythrin detection agent (50 µl) was added to each sample and incubated in the dark at 4°C for 2 h. Thereafter, the samples were supplemented with 1 ml washing buffer and centrifuged at 200 × *g* for 5 min. The supernatant was discarded, and the cell pellet was resuspended in washing buffer (300 µl). The cytokine levels were measured using a FACS Canto II (BD Biosciences) and analyzed with FCAP Array™ software (version 3, BD Biosciences).

### CD8^+^ T Cell Depletion

CD8^+^ T cells were depleted by the injection of rat anti-mouse CD8 β (clone 53.5.8) (BioXell, West lebanon, NH, USA). Depletion was performed by i.p. injection of 500 µg of purified anti-CD8^+^ T cell antibody starting 3 days before infection. Control mice were treated with i.p. injection of 500 µg of non-immune rat IgG. The depletion was assessed by flow cytometry.

### Statistics

Statistical differences for survival curves were analyzed by log-rank test. Statistical differences between groups were analyzed using the two-tailed Student’s *t*-test. All experiments were performed at least twice. *P* values of <0.05 were considered significant.

## Author Contributions

US, WS, JK, MR, and XW conducted the experiments. MN and KM provided experimental techniques and material. DS and GN analyzed data and wrote the manuscript.

## Conflict of Interest Statement

The authors declare that the research was conducted in the absence of any commercial or financial relationships that could be construed as a potential conflict of interest.

## Supplementary Material

Figure S1 **Increased levels of interferon (IFN)-γ in the serum of *Cyld*^−/−^ mice**. The serum concentrations of interleukin (IL)-10, IL-17, tumor necrosis factor, IFN-γ, IL-6, IL-4, and IL-2 were determined in *Plasmodium berghei* ANKA (*PbA*)-infected WT (open symbols) and *Cyld*^−/−^ mice (black symbols) by a cytometric bead assay at day 7 postinfection. IFN-γ levels were significantly increased in *Cyld*^−/−^ mice compared to WT mice. **p* < 0.05 (two-tailed Student’s *t*-test). Six mice were analyzed per group, and representative data are shown.

Figure S2 **Enhanced *Plasmodium berghei* ANKA (PbA)-specific CD8^+^ T cell response in the blood of *Cyld*^−/−^ mice after blood stage infection**. **(A)** Relative numbers of *Pb*A antigen-specific granzyme B-producing CD8^+^ T cells from uninfected (day 0) and *Pb*A-infected WT and *Cyld*^−/−^ mice (day 7). **(B)** Absolute numbers of CD3^+^CD8^+^ T cells in the blood of uninfected (day 0) and *Pb*A-infected WT and *Cyld*^−/−^ mice (day 7) (*n* = 6 each). **(C)** Absolute number of interferon-γ-producing CD8^+^ T cells in the blood of uninfected (day 0) and *Pb*A-infected mice (day 7) after *ex vivo* restimulation with GAP-50 peptide. **p* < 0.05 (two-tailed Student’s *t*-test). Data from one of three independent experiments are shown.

Figure S3 **Reduced *Plasmodium berghei* ANKA (PbA)-specific CD8^+^ T cell response in the brain of *Cyld*^−/−^ mice after blood-stage infection**. **(A,B)** Absolute numbers of CD3^+^ CD8^+^ T cells **(A)** and interferon-γ-producing CD8^+^ T cells **(B)** after *ex vivo* restimulation with GAP-50 peptide in the brains of uninfected (day 0) and *Pb*A blood stage-infected mice (day 7) in WT and *Cyld*^−/−^ mice (*n* = 6 each). **p* < 0.05 (two-tailed Student’s *t*-test). Data from one of three independent experiments are shown.

Figure S4 **Efficient depletion of CD8^+^ T cell by monoclonal antibody treatment**. C57BL/6 *Cyld*^−/−^ and WT (*n* = 6) mice were either treated with anti-CD8 antibody or rat IgG starting 3 days before intraperitoneal infection with 1 × 10^6^ *Plasmodium berghei* ANKA-infected red blood cells. At day 7 postinfection, efficiency of T cell depletion was determined by flow cytometry. Data show relative numbers of anti-CD3- and anti-CD8-stained blood cells of representative mice (*n* = 3 per group).

Figure S5 **Reduced chemokine receptor expression by CD8^+^ T cell in the spleen of *Cyld*^−/−^ mice after blood-stage infection**. **(A,C)** Histogram overlays show data for CCR1 **(A)** and CCR5 **(C)** expression in CD3^+^ CD8^+^ T cells of uninfected (day 0) and day 7 postinfection (day 7) WT and *Cyld*^−/−^ mice. **(B,D)** The mean fluorescence intensity of CCR1 **(B)** and CCR5 **(D)** is shown for WT and *Cyld*^−/−^ mice at the indicated time points. Specific staining for WT mice is shown in light gray and for *Cyld*^−/−^ mice in dark gray. Data from one of two experiments (*n* = 6) are shown.
