# Supplementary figures and images for: The Deubiquitinating Enzyme Cylindromatosis Dampens CD8+ T Cell Responses and Is a Critical Factor for Experimental Cerebral Malaria and Blood–Brain Barrier Damage
Source: Front Immunol. 2017 Feb 1;8:27. doi: 10.3389/fimmu.2017.00027 (PMC5285367; doi:10.3389/fimmu.2017.00027)

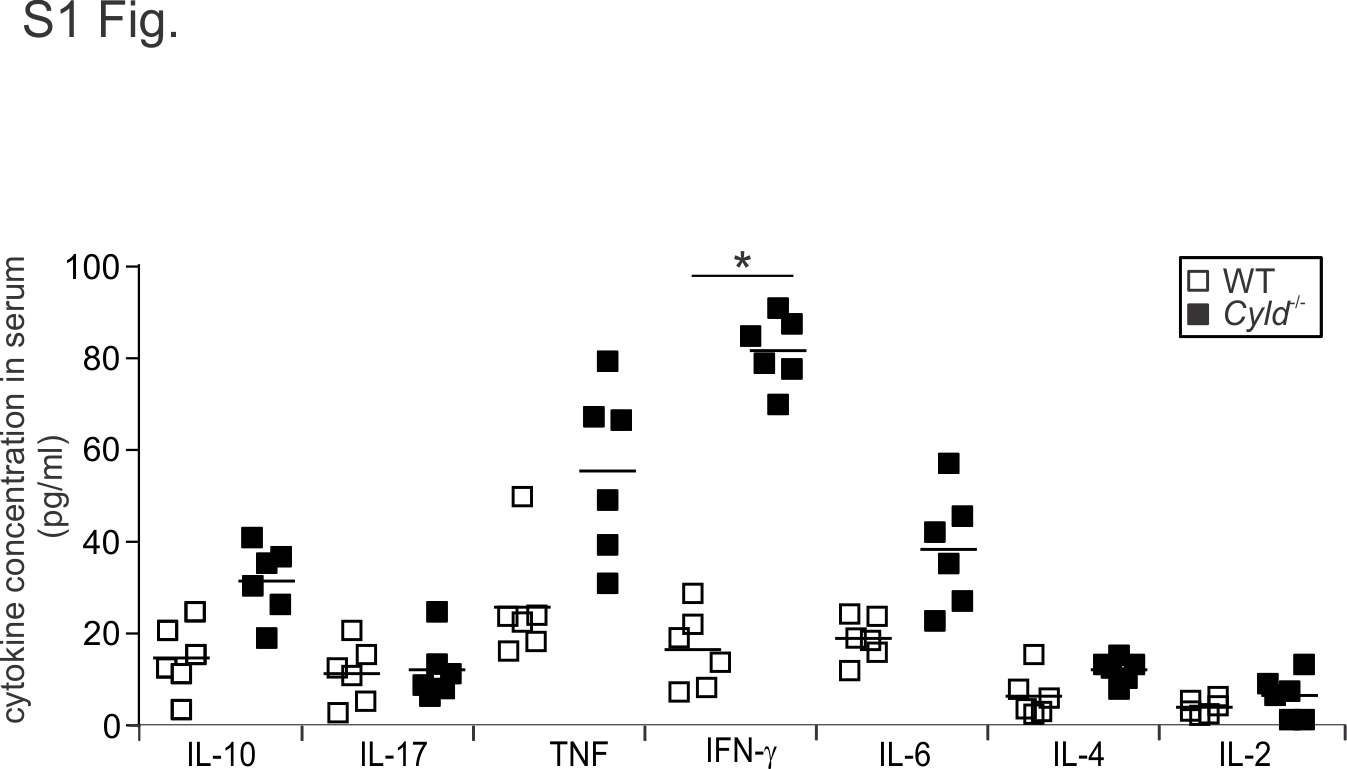

Supplement: Figure S1 — Increased levels of interferon (IFN)-γ in the serum of Cyld−/− mice. The serum concentrations of interleukin (IL)-10, IL-17, tumor necrosis factor, IFN-γ, IL-6, IL-4, and IL-2 were determined in Plasmodium berghei ANKA (PbA)-infected WT (open symbols) and Cyld−/− mice (black symbols) by a cytometric bead assay at day 7 postinfection. IFN-γ levels were significantly increased in Cyld−/− mice compared to WT mice. *p < 0.05 (two-tailed Student’s t-test). Six mice were analyzed per group, and representative data are shown. [file Image_1.TIF]

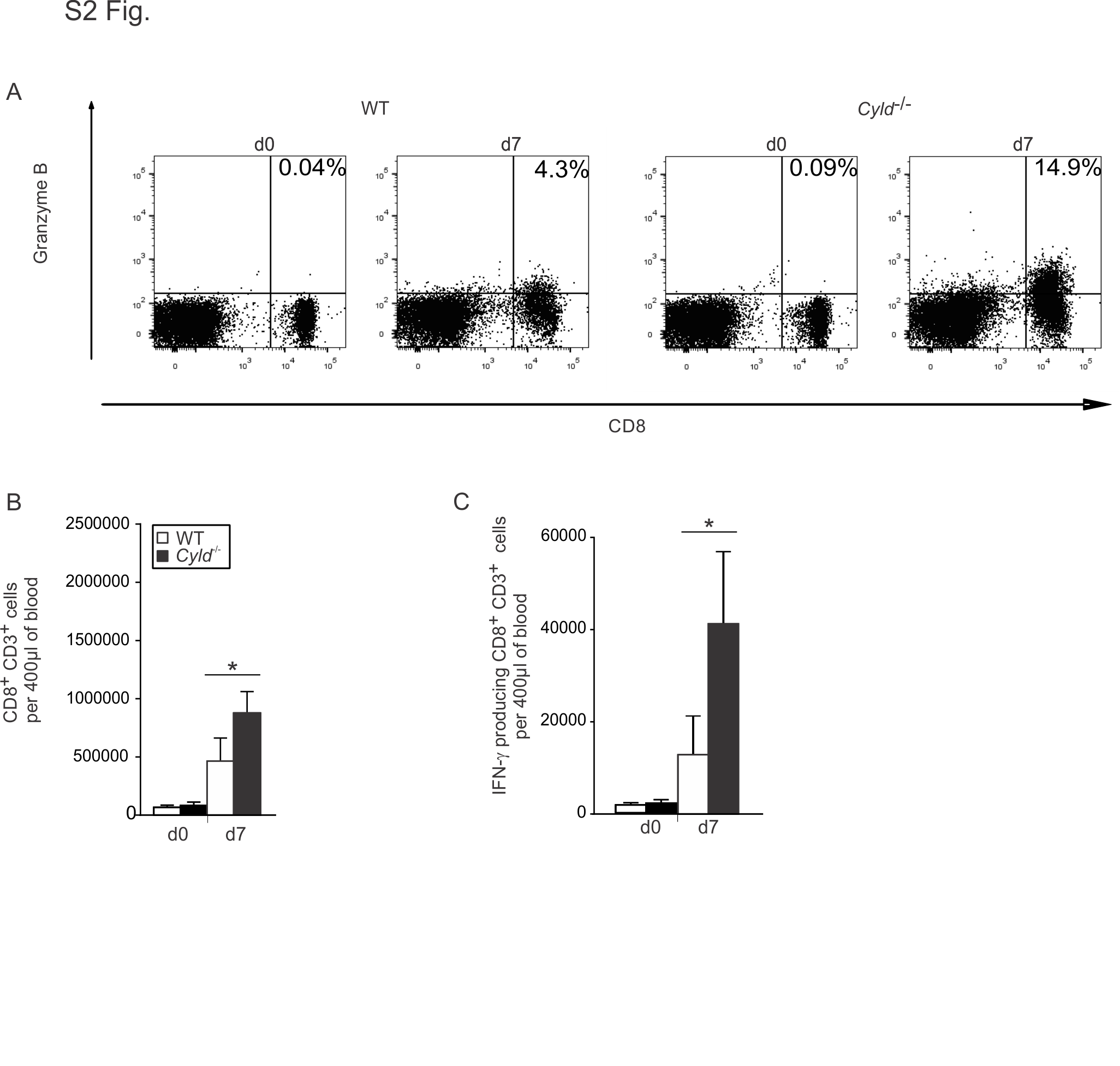

Supplement: Figure S2 — Enhanced Plasmodium berghei ANKA (PbA)-specific CD8+ T cell response in the blood of Cyld−/− mice after blood stage infection. (A) Relative numbers of PbA antigen-specific granzyme B-producing CD8+ T cells from uninfected (day 0) and PbA-infected WT and Cyld−/− mice (day 7). (B) Absolute numbers of CD3+CD8+ T cells in the blood of uninfected (day 0) and PbA-infected WT and Cyld−/− mice (day 7) (n = 6 each). (C) Absolute number of interferon-γ-producing CD8+ T cells in the blood of uninfected (day 0) and PbA-infected mice (day 7) after ex vivo restimulation with GAP-50 peptide. *p < 0.05 (two-tailed Student’s t-test). Data from one of three independent experiments are shown. [file Image_2.TIF]

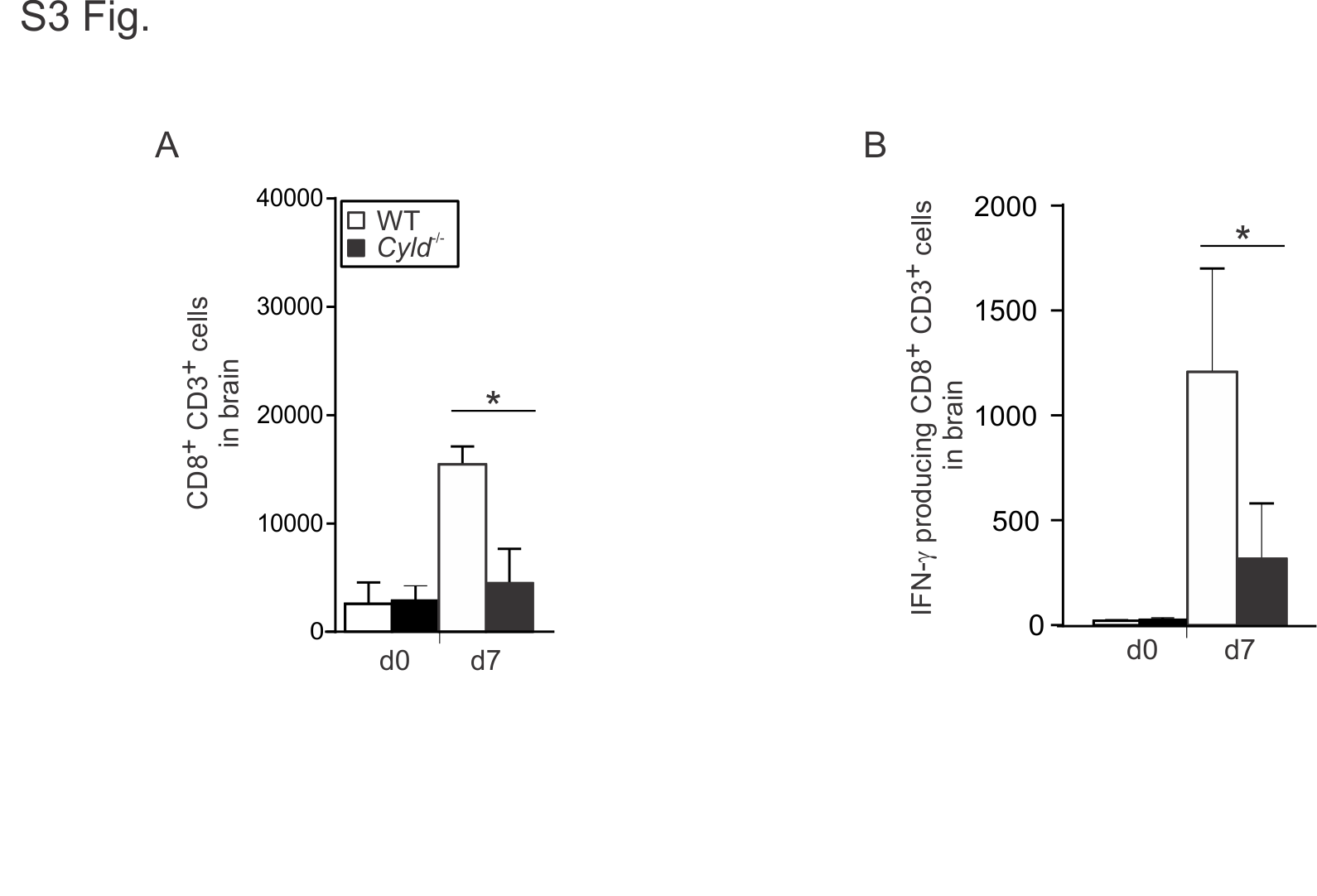

Supplement: Figure S3 — Reduced Plasmodium berghei ANKA (PbA)-specific CD8+ T cell response in the brain of Cyld−/− mice after blood-stage infection. (A,B) Absolute numbers of CD3+ CD8+ T cells (A) and interferon-γ-producing CD8+ T cells (B) after ex vivo restimulation with GAP-50 peptide in the brains of uninfected (day 0) and PbA blood stage-infected mice (day 7) in WT and Cyld−/− mice (n = 6 each). *p < 0.05 (two-tailed Student’s t-test). Data from one of three independent experiments are shown. [file Image_3.TIF]

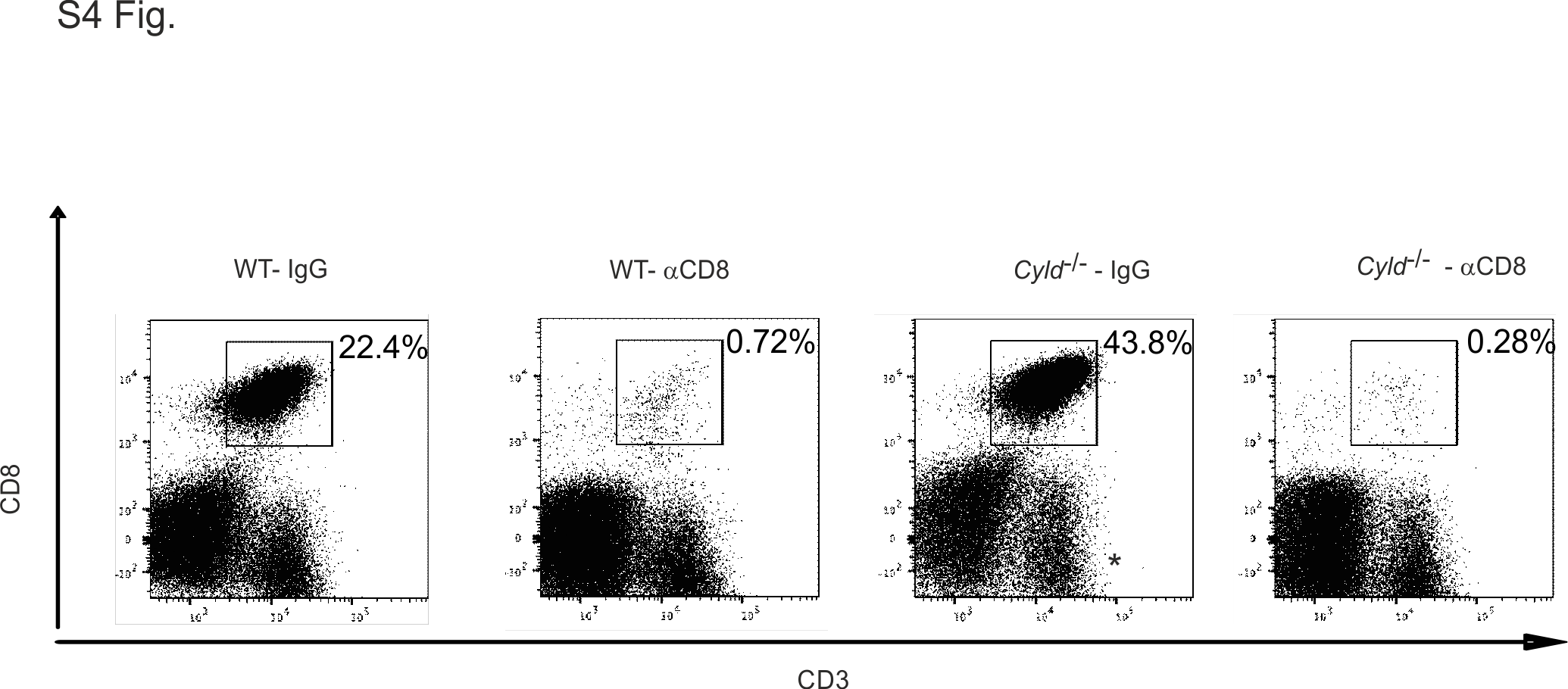

Supplement: Figure S4 — Efficient depletion of CD8+ T cell by monoclonal antibody treatment. C57BL/6 Cyld−/− and WT (n = 6) mice were either treated with anti-CD8 antibody or rat IgG starting 3 days before intraperitoneal infection with 1 × 10^6^ Plasmodium berghei ANKA-infected red blood cells. At day 7 postinfection, efficiency of T cell depletion was determined by flow cytometry. Data show relative numbers of anti-CD3- and anti-CD8-stained blood cells of representative mice (n = 3 per group). [file Image_4.TIF]

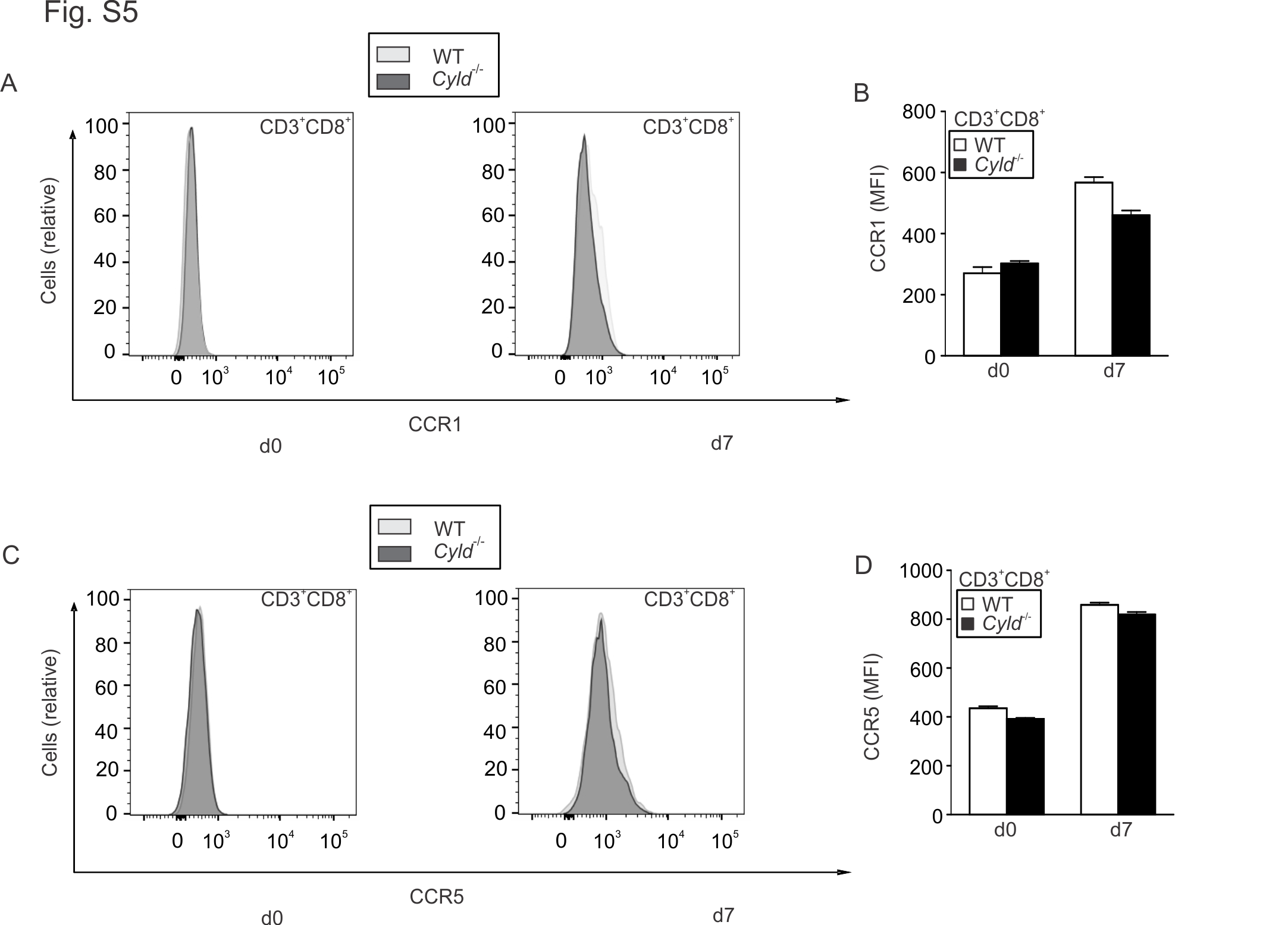

Supplement: Figure S5 — Reduced chemokine receptor expression by CD8+ T cell in the spleen of Cyld−/− mice after blood-stage infection. (A,C) Histogram overlays show data for CCR1 (A) and CCR5 (C) expression in CD3+ CD8+ T cells of uninfected (day 0) and day 7 postinfection (day 7) WT and Cyld−/− mice. (B,D) The mean fluorescence intensity of CCR1 (B) and CCR5 (D) is shown for WT and Cyld−/− mice at the indicated time points. Specific staining for WT mice is shown in light gray and for Cyld−/− mice in dark gray. Data from one of two experiments (n = 6) are shown. [file Image_5.TIF]
